# Genotype–Phenotype Associations in Phelan–McDermid Syndrome: Insights into Novel Genes Beyond *SHANK3*

**DOI:** 10.3390/ijms26104653

**Published:** 2025-05-13

**Authors:** Julian Nevado, Blanca Escalada, Yolanda Muñoz-GªPorrero, Carmen Adan, Jair Tenorio-Castaño, Pablo Daniel Lapunzina

**Affiliations:** 1 Instituto de Genética Médica y Molecular (INGEMM)-IdiPAZ, Hospital Universitario La Paz, Paseo de la Castellana 261, 28046 Madrid, Spain; blancaescalada@gmail.com (B.E.); ymunozg@salud.madrid.org (Y.M.-G.); carmen.adan@salud.madrid.org (C.A.); jaira.tenorio@salud.madrid.org (J.T.-C.); or plapunzina@ciberer.es (P.D.L.); 2 CIBERER—Centro de Investigación Biomédica en Red de Enfermedades Raras, ISCIII, 28029 Madrid, Spain; 3 ITHACA-European Reference Network-Hospital La Paz, 28046 Madrid, Spain

**Keywords:** Phelan–McDermid syndrome (PMS), *SHANK3*, 22q13.33 chromosomal region, genotype–phenotype correlation, haploinsufficiency

## Abstract

Phelan–McDermid syndrome (PMS; #MIM: 606232) is a rare neurodevelopmental disorder primarily caused by the haploinsufficiency of the *SHANK3* gene, most often due to deletions encompassing the gene or single nucleotide variants within it. Individuals with PMS display a wide range of clinical abnormalities and considerable genetic heterogeneity. This study aims to investigate genotype–phenotype correlations in a cohort of 213 individuals with PMS and to identify novel candidate genes, beyond *SHANK3*, that may contribute to the syndrome’s diverse clinical manifestations. Unsupervised clustering based on deletion size and Global Functional Assessment of the Patient (GFAP, previously described and developed by our group), along with additional analytical approaches, were employed to explore genotype–phenotype relationships. Deletion size within the 22q13.3 region emerged as a major determinant of phenotype, with larger deletions associated with more severe global functional impairment. Furthermore, *CERK*, *TBC1D22A*, *CELSR1*, and *GRAMD4* were identified as candidate genes within 22q13.3, potentially contributing to core PMS phenotypes, and their putative interactions were explored. Our findings support the central role of *SHANK3* in PMS, while also indicating that it does not account for the full phenotypic spectrum. This study underscores the variable impact of distinct genetic alterations in PMS and proposes additional loci implicated in its pathogenesis. These insights may inform future therapeutic strategies, emphasizing the importance of patient stratification and precision medicine.

## 1. Introduction

Phelan–McDermid syndrome (PMS; #MIM: 606232) is a rare genetic disorder. In Europe, a disease is classified as rare when its prevalence is ≤1 in 2000 individuals. Based on local data, we estimate a national prevalence of 4 × 10^−4^ per 10,000 inhabitants [1], which is lower than the previously reported, 1 in 20,000 [2], suggesting underdiagnosis. PMS is a neurodevelopmental disorder caused by haploinsufficiency of the *SHANK3* gene, resulting either from deletions at 22q13.33 [3,4] or from pathogenic or likely pathogenic variants [3,5]. *SHANK3* encodes a scaffolding protein essential to the postsynaptic density of excitatory neurons, playing a critical role in synaptic signaling, plasticity, and neural circuit formation [6].

PMS manifests with a broad spectrum of neurological, behavioral, and physical anomalies [7,8], including autism spectrum disorder (ASD) [9,10], intellectual disability [4], and schizophrenia [11]. Additional features include hypotonia, high pain tolerance, impulsivity, repetitive behaviors, developmental regression, and continuous crying [12]. Structural brain abnormalities, gastrointestinal disturbances, and renal malformations are also commonly observed [3,4,13]. Although *SHANK3* haploinsufficiency is central to the disorder, other genes have been previously implicated in the PMS phenotype. For instance, *TFC20* has been associated with intellectual disability and ASD [14], and *CELSR1* has been linked to lymphedema [15,16,17].

Patients with deletions are more common than those carrying pathogenic *SHANK3* single nucleotide variants (SNVs) (International PMS Registry; [3,4]). These deletions typically arise through non-allelic homologous recombination, unbalanced translocations, or other genomic rearrangements, such as ring chromosome 22 [3,8]. Most deletions occur de novo, although some cases result from parental mosaicism or balanced rearrangements [3]. Deletion sizes range from 12 kb to 10.3 Mb [3] and can be detected using CGH or SNP arrays, FISH, MLPA [3], or karyotyping for larger deletions (>4–5 Mb) [18].

## 2. Results

### 2.1. “Functional (GFAP)” Analyses Between the Different Groups of Patients with PMS

**Figure 1:**
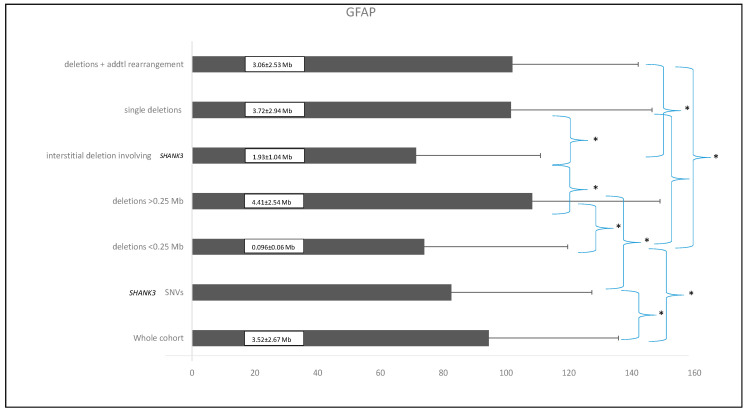
Comparison of the different patient subgroups based on their mean GFAP values (arbitrary units) and standard deviations. Asterisks (*) indicate statistically significant differences (*p* ≤ 0.01). Numbers within the black bars represent the mean ± standard deviation of the deletion size for each corresponding group.

The global functional assessment of the patients (GFAP), as previously described [3], was applied in this cohort to stratify individuals into distinct groups: small deletions (≤0.25 Mb), deletions > 0.25 Mb, *SHANK3* SNVs, isolated deletions, deletions with additional genomic rearrangements, and interstitial deletions. ANOVA analysis revealed statistically significant functional differences among these groups (F = 7.133; df = 5; *p* < 0.001), as illustrated in Figure 1.

**Figure 2:**
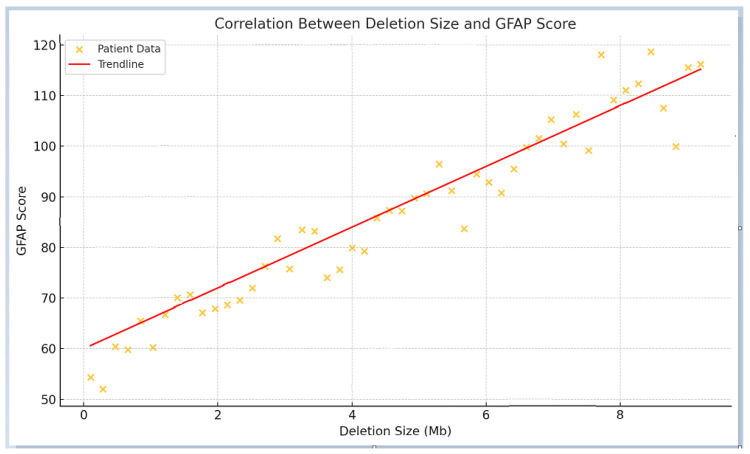
Correlation between deletion size and GFAP score. The scatter plot illustrates a positive correlation between deletion size and GFAP scores, with larger deletions corresponding to more severe phenotypes and higher GFAP values. The red trend line highlights the overall linear relationship. The arrow denotes a potential inflection point beyond which functional outcomes appear to worsen more markedly.

The mean GFAP score for the entire cohort was 94.5 ± 41.3 arbitrary units. Patients with smaller deletions (≤0.25 Mb) had a mean GFAP score of 73.98 ± 45.7, compared to 108.34 ± 40.67 in those with deletions >0.25 Mb, demonstrating a highly significant statistical difference (*p* < 0.0001). As expected, individuals with smaller deletions exhibited a functional profile closely resembling that of patients with *SHANK3* SNVs, suggesting overlapping pathogenic mechanisms. Overall, patients with smaller deletions presented with fewer comorbidities and lower GFAP scores, indicating better functional outcomes compared to those with larger deletions. In contrast, individuals with larger deletions were associated with higher GFAP scores, reflecting increased clinical severity, including profound intellectual disability, pronounced hypotonia, and significant behavioral disturbances. These findings support the consideration of deletion size as a key determinant of clinical severity, with larger deletions consistently correlating with higher GFAP scores. Figure 2 illustrates the average GFAP scores across different deletion size categories, highlighting that deletions exceeding 2.7 Mb are unequivocally associated with the highest GFAP scores, indicative of more severe functional impairment.

Interestingly, interstitial deletions involving *SHANK3* exhibited statistically significant differences in GFAP scores when compared to cases with additional genomic rearrangements, isolated deletions, or deletions larger than 0.25 Mb. In contrast, interstitial deletions within 22q13.3 that do not include *SHANK3* (*n* = 3) also yielded GFAP scores, with a mean value of 45.3 ± 16.8—the lowest among the groups analyzed. Notably, some of these individuals, despite the absence of *SHANK3* involvement, presented clinical features reminiscent of PMS.

### 2.2. Genotype–Phenotype Analyses

#### 2.2.1. Pearson’s Correlation Analyses (Continuous Variables)

Pearson’s correlation coefficient was used to assess the strength and direction of relationships between continuous variables, including deletion size, GFAP scores, and the number of affected genes. The results are summarized in Table 1. Larger deletions were positively correlated with higher GFAP scores, indicating more severe “functional” outcomes. As anticipated, an increase in the number of deleted genes was also associated with elevated GFAP scores, suggesting that the loss of multiple functional genes across various regions contributes to increased phenotypic severity. Consistent with previous observations [3], larger deletions tended to be diagnosed earlier, likely due to their more overt clinical presentation. Furthermore, deletion size showed a strong correlation with the number of OMIM genes lost, supporting the hypothesis that PMS with large deletions may represent a true contiguous gene syndrome, wherein the cumulative effect of multiple gene losses contributes to the clinical phenotype (Table 1).

#### 2.2.2. Spearman’s Rank Correlation Analyses (Categorical Variables)

Spearman’s correlation was employed to assess associations between ordinal phenotypic traits and both deletion size and GFAP scores. Notably, larger deletions were inversely correlated with expressive language abilities (r = −0.421; *p* < 0.0001), comprehension skills (r = −0.266; *p* < 0.0001), and sphincter control (r = −0.277; *p* < 0.0001). Conversely, deletion size was positively correlated with the prevalence and severity of hypotonia (r = 0.304; *p* < 0.0001), growth anomalies (r = 0.227; *p* = 0.002), toe syndactyly (r = 0.223; *p* = 0.002), renal–urological abnormalities (r = 0.197; *p* = 0.007), lymphedema (r = 0.277; *p* < 0.0001), and large/fleshy hands (r = 0.266; *p* < 0.001). Additional traits—including seizures, strabismus (inversely), prominent ears, full/puffy eyelids, and hair-pulling—demonstrated unidirectional statistical significance (see Table S1, Supplemental Data).

Regarding associations with GFAP scores, we observed significant positive correlations with seizures (r = 0.207; *p* < 0.0001), developmental regression (r = 0.424; *p* < 0.0001), behavioral anomalies (r = 0.384; *p* < 0.0001), and high pain threshold (r = 0.358; *p* < 0.0001). Other notable correlations included core dysmorphic traits (e.g., long eyelashes, full brow, bulbous nose, wide nasal bridge, prominent ears) and various comorbidities such as renal abnormalities, gastrointestinal disturbances, hearing impairments, sleep disorders, and ophthalmological anomalies (see Table S1, Supplemental Data).

#### 2.2.3. Analysis of Unsupervised Clusters by Deletion Size (Ward’s Method)

**Figure 3:**
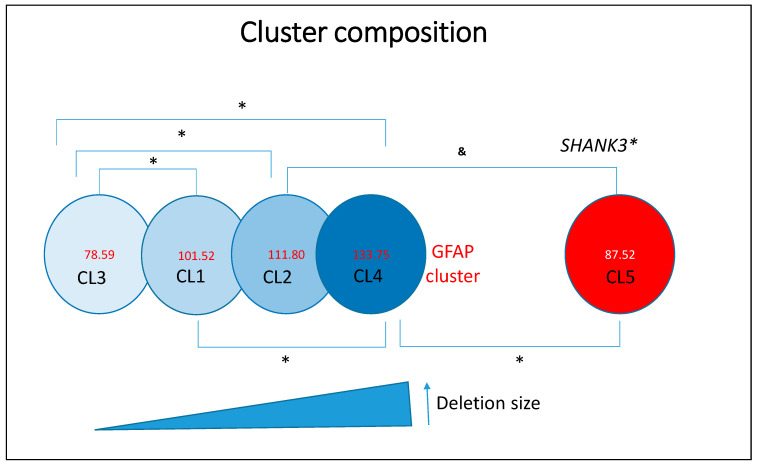
Representation of deletion-based clusters and SHANK3 variants as a function of GFAP scores. Asterisks (*) indicate statistically significant differences (*p* < 0.05). The ampersand symbol (&) denotes a trend toward significance that does not reach the conventional threshold of statistical significance (*p* ≥ 0.05).

Unsupervised clustering was conducted using Ward’s hierarchical method, based on deletion size and associated phenotypic features (see Figure 3). The optimal number of clusters (*n* = 4) was determined using the Bayesian Information Criterion (BIC) and Akaike Information Criterion (AIC). Four primary clusters were identified, and a fifth cluster (Cluster 5, *SHANK3* SNVs) was added to complete the comparative analysis. These clusters revealed distinct genetic and phenotypic profiles: Cluster 1 (CL1), mean deletion size = 3.054 ± 1.01 Mb (81 individuals); mean GFAP = 101.52 ± 39.8; Cluster 2 (CL2), mean deletion size = 6.29 ± 0.78 Mb (34 individuals); mean GFAP = 111.80 ± 37.96; Cluster 3 (CL3), mean deletion size = 0.246 ± 0.40 Mb (51 individuals); mean GFAP = 78.59 ± 46.93; Cluster 4 (CL4), mean deletion size = 8.48 ± 0.66 Mb (24 individuals); mean GFAP = 133.75 ± 32.60 and Cluster 5 (CL5, *SHANK3* SNVs), no deletion (21 individuals); mean GFAP = 87.52 ± 22.8.

Frequency data for the various clinical variables across clusters are presented in Table S2 of the Supplemental Data. In brief, Cluster 1 (moderate deletions) comprises individuals with moderate functional impairments, characterized by a combination of developmental delays and behavioral features. Cluster 2 (large deletions) includes individuals with severe phenotypes, notably marked by profound hypotonia and significant cognitive delays. Cluster 3 (small deletions) consists of individuals with the mildest functional impairments, reflected by lower GFAP scores and a higher frequency of early independent walking and preserved language abilities.

As anticipated, Cluster 4 (very large deletions) represents individuals with the most severe clinical presentations, as indicated by the highest GFAP scores and elevated frequencies of seizures, renal anomalies, and macrocephaly. Cluster 5 (*SHANK3* SNVs) includes individuals with comparatively milder phenotypes, functionally similar to those in Cluster 3. Overall, deletion size emerges as a key determinant of phenotypic severity in PMS, influencing a broad range of clinical features including non-fluent language, seizures, macrocephaly, dolichocephaly, strabismus, deep-set eyes, full/puffy eyelids, full eyebrows, wide nasal bridge, bulbous nose, auditory abnormalities, full/puffy cheeks, syndactyly, dysplastic nails, large/fleshy hands, ophthalmological anomalies, nephrourological abnormalities, obesity, lymphedema, uncontrolled laughter, hair-pulling, and altered growth trajectories. The frequency of these features increases with deletion size, which in turn correlates with both elevated GFAP scores and the number of deleted genes, including OMIM-annotated genes.

#### 2.2.4. ANOVA Analysis-Comparison of GFAP Scores Across Clusters

A one-way ANOVA was conducted to compare GFAP scores across the five clusters, revealing statistically significant differences (F = 7.061; degrees of freedom = 5; *p* < 0.001). Specifically, individuals in Cluster 3 (small deletions) exhibited significantly better functional outcomes compared to those in Clusters 2 and 4 (larger deletions). No statistically significant difference was observed between Clusters 3 and 5 (*p* = 0.2), indicating that the phenotype associated with small deletions may closely resemble that of individuals with *SHANK3* SNVs. Similarly, no significant difference was found between Clusters 2 and 4 (*p* = 0.4), suggesting that larger deletions consistently confer poor functional outcomes.

#### 2.2.5. Chi-Square Test (For Categorical Variables)

Chi-square analyses were performed to assess associations between categorical phenotypic traits and the different genetic groups represented by the clusters described above. In addition, the application of χ^2^ tests followed by post hoc analyses revealed several statistically significant differences (*p* < 0.001), as detailed in Table 2 and Table 3.

- By Cluster. Table 2 presents statistically significant associations between clusters and various categorical variables. In most cases, the observed data corroborated previously reported frequency patterns across clusters. Notable differences were identified in cognitive and motor domains, as well as in specific comorbidities and dysmorphic features (see Table 2). Pairwise comparisons between clusters are further detailed in Table S3 of the Supplemental Data. For example, seizures were significantly more prevalent in Clusters 2 and 4 (χ^2^ = 21.42, *p* < 0.001). Regarding language ability, Clusters 3 and 5 showed better outcomes, with a significantly higher proportion of individuals demonstrating preserved language skills (χ^2^ = 14.89, *p* = 0.001). Additionally, cranial size abnormalities—namely microcephaly and macrocephaly—were differentially associated with cluster assignment based on deletion size, with larger deletions linked to a higher prevalence of macrocephaly and a lower prevalence of microcephaly. Table 2 presents only those variables showing statistically significant differences among the clusters.
- By Genetic Groups (Table 3). As shown in Table 3, significant differences in language abilities were observed between individuals with large deletions (>0.25 Mb) and those carrying *SHANK3* SNVs (χ^2^ = 26.345; degrees of freedom = 2; *p* < 0.001). Additionally, renal abnormalities and lymphedema were significantly more prevalent among patients with deletions encompassing the *CELSR1* and *GRAMD4* genes (χ^2^ = 18.734; degrees of freedom = 2; *p* < 0.001). Hypotonia and seizures were also significantly more frequent in individuals with larger deletions, particularly those affecting *TBC1D22A* and *CELSR1* (χ^2^ = 18.734; degrees of freedom = 2; *p* < 0.001). Comparisons among other groups are further detailed in Table S4 of Supplemental Data.

### 2.3. Logistic Regression Analysis

To investigate the predictive value of genetic variables on clinical outcomes, several logistic regression models were constructed and evaluated. Two key models are highlighted below.

(i) Prediction of speech delay (defined as the inability to form sentences): Independent variables included deletion size, presence of *SHANK3* SNVs, and the number of deleted OMIM-annotated genes. The analysis revealed that for every 1 Mb increase in deletion size, the likelihood of significant speech delay increased by 85%. The odds ratio (OR) for deletion size was 1.85 (95% CI: 1.40–2.43; *p* < 0.001).
(ii) Prediction of hypotonia: Independent variables included deletion size and GFAP score. The results indicated that larger deletions were significantly associated with an increased risk of hypotonia. The odds ratio for deletion size was 2.31 (95% CI: 1.75–3.12; *p* < 0.001).

### 2.4. Candidate Gene Analysis: GFAP Comparison Based on Clinical Status

To explore the potential involvement of candidate genes, we compared GFAP scores between individuals with and without specific clinical features. Binary variables (“1/Yes” vs. “0/No”) were analyzed using independent-samples Student’s *t*-tests, following Levene’s test for equality of variances. For variables with more than two categories, ANOVA was used. Variables showing statistically significant differences in GFAP scores based on clinical status are presented in Table 4.

**Figure 4:**
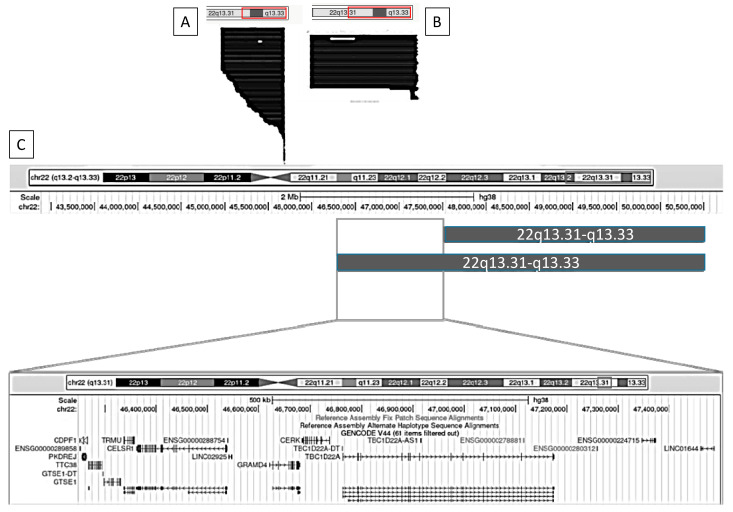
Graphical representation using the UCSC Genome Browser of patients with and without nephro-urological alterations. (**A**) Patients without nephro-urological alterations; (**B**) patients with nephro-urological alterations; (**C**) comparison of the haploinsufficiency regions between groups A and B. Genomic coordinates are based on the GRCh38 (hg38) reference assembly.

**Figure 5:**
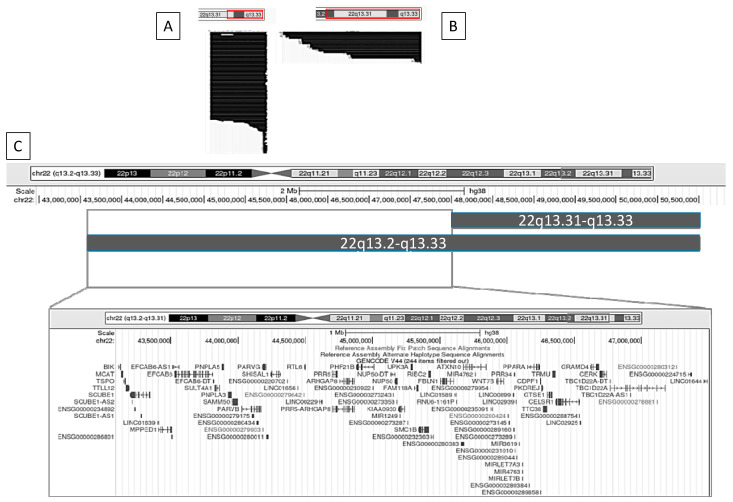
Graphical representation using the UCSC Genome Browser of patients with and without lymphedema. (**A**) Patients without lymphedema; (**B**) patients with lymphedema; (**C**) comparison of the haploinsufficiency regions between groups A and B. Genomic coordinates are based on the GRCh38 (hg38) reference genome.

Figure 4 and Figure 5 illustrate, as examples, two clinical variables that demonstrated statistically significant differences in GFAP scores. For each, we visualized the data using the UCSC Genome Browser (https://genome.ucsc.edu/ (accessed on 17 February 2025)) by grouping individuals based on the presence or absence of the clinical feature and plotting the corresponding median deletion sizes. This schematic approach facilitates the comparison of genomic intervals associated with differential clinical outcomes.

#### 2.4.1. Nephro-Urological Alterations

We identified a set of genes that differ most prominently between individuals with and without nephro-urological alterations. These include *TBC1D22A*, *CERK*, *GRAMD4*, *CELSR1*, *TRMU*, *CDPF1*, *PKDREJ*, *TTC38*, and *GTS1*.

#### 2.4.2. Lymphedema

We identified a set of genes that differ most prominently between individuals with and without lymphedema. These include *TBC1D22A*, *CERK*, *GRAMD4*, *CELSR1*, *TRMU*, *CTSE1*, *CDPF1*, *TTC38*, *PKDREJ*, *PPARA*, *WNT7B*, *ATXN10*, *FBLN1*, *RIBC2*, *SMC1B*, *FAM118A*, *UPK3A*, *NUP50*, *KIAA0930*, *PHF21B*, *ARHGAP8*, *PRR5-ARHGAP8*, *PRR5*, *RTL6*, *PARVG*, *SHISAL1*, *PARVB*, *SAMM50*, *PNPLA3*, *PNPLA5*, *SULT4A1*, *EFCAB6*, *MPPED1*, *SCUBE1*, *TTLL12*, *TSPO*, *MCAT*, *and BIK.*

The remaining clinical features that showed statistically significant associations in Table 4 are depicted in Figures S1–S9 of the Supplemental Data. Across the comparisons of all positive clinical conditions, we observed a recurrent and consistent haploinsufficient region in individuals exhibiting the different clinical traits. This shared region encompasses four key genes: *TBC1D22A*, *CERK*, *GRAMD4*, and *CELSR1*.

### 2.5. Analysis of Haploinsufficiency in Deleted Genes Within 22q13.31–22q13.33

To identify and characterize potential haploinsufficient regions within the 22q13.31–22q13.33 interval (8.3 Mb), we developed a composite haploinsufficiency score based on the sum of outputs from five established haploinsufficiency prediction algorithms (see Section 4). This yielded an overall score for each gene, visualized using a color-coded scheme and detailed in Table 5 and Table 6 below, as well as Table S5 of the Supplemental Data. These tables include the gene name, genomic coordinates, and calculated haploinsufficiency score. Gene functions, annotated through Gene Ontology (GO), are further illustrated. Based on the scores, genes were categorized as follows: Red: Score > 30, classified as highly haploinsufficient (Table 5); Yellow: Score > 20, classified as moderately haploinsufficient (Table 6); Orange: Score 10–20, classified as weakly haploinsufficient (Table S5a, Supplemental Data); Green: Score < 10, classified as very weakly haploinsufficient (Table S5b, Supplemental Data).

**Figure 6:**
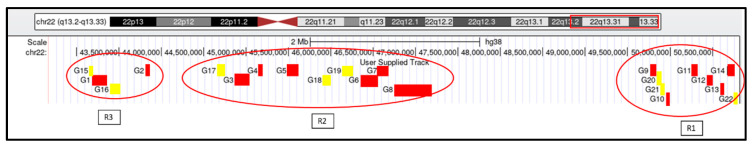
Visualization of genes and genomic regions within 22q13.31–q13.33 identified as highly or moderately susceptible to haploinsufficiency. Genomic coordinates are based on the GRCh38 (hg38) reference assembly. Based on the scores, genes were categorized as follows: Red: Score > 30, classified as highly haploinsufficient; Yellow: Score > 20, classified as moderately haploinsufficient.

Based on the analysis of the previous tables, a customized track was created in the UCSC Genome Browser by entering the genomic coordinates of genes classified as highly and moderately haploinsufficient—highlighted in red and yellow, respectively. This visualization was used to identify potential haploinsufficient regions relevant to Phelan–McDermid syndrome within our cohort. As a result, three distinct regions of significant haploinsufficiency were identified and are illustrated in Figure 6.

The first haploinsufficient region (R1) comprises the following genes: G9 = *BRD1*, G10 = *PIM3*, G11 = *PLXNB2*, G12 = *SBF1*, G13 = *MAPK8IP2*, G14 = *SHANK3*, G20 = *ZBED4*, G21 = *ALG12*, and G22 = *RABL2B*. The second region (R2) includes G3 = *PHF21B*, G4 = *NUP50*, G5 = *FBLN1*, G6 = *CELSR1*, G7 = *GRAMD4*, G8 = *TBC1D22A*, G17 = *PRR5*, G18 = *WNT7B*, and G19 = *PPARA.* A third region (R3) is composed of G1 = *SCUBE1*, G2 = *SULT4A1*, G15 = *TTLL12*, and G16 = *MPPED1*. Region 1 includes the *SHANK3* gene—considered the key gene in PMS—along with several neighboring genes. Region 2 aligns with the set of genes previously identified through regional comparisons (Section 4.3), particularly *TBC1D22A*, *CERK*, *GRAMD4*, and *CELSR1.* Interestingly, the mean haploinsufficiency score of genes in Region 2 (*n* = 9) is equivalent to that in Region 1 (*n* = 9), and notably higher than that of Region 3 (*n* = 4).

#### Analysis of Interaction Pathways Among Identified Haploinsufficient Genes

**Figure 7:**
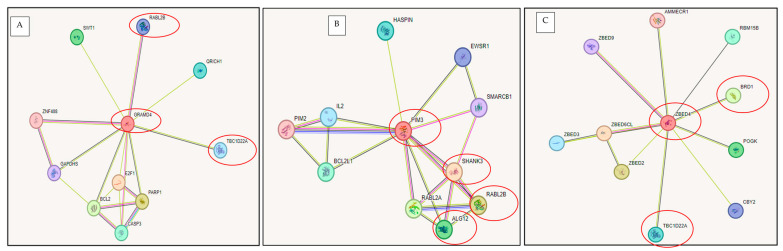
Interaction networks of selected haploinsufficient genes generated using STRING (https://string-db.org/ (accessed on 16 February 2015)). (**A**) Proteins interacting with GRAMD4; (**B**) Proteins interacting with PIM3; (**C**) Proteins interacting with ZBED4.

To examine the potential functional interactions among the haploinsufficient genes within Regions R1–R3, we analyzed known and predicted pathways using the STRING database (https://string-db.org/ (accessed on 17 February 2025)). Interactions were identified based on experimental data, text mining, and gene co-expression. The resulting interaction networks are illustrated in Figure 7A–C.

Gene–gene associations were identified both within and between the defined regions. Notably, inter-regional connections between genes at Regions R1 and R2 were observed through *GRAMD4*, *TBC1D22A*, and *RABL2B* (Figure 7A), while intra-regional associations within Region 1 were identified among *PIM3*, *SHANK3*, *RABL2B*, and *ALG12* (Figure 7B). Additionally, connections were observed involving *ZBED4*, *BRD1*, and *TBC1D22A*, linking Regions R1 and R2, as well (Figure 7C).

**Figure 8:**
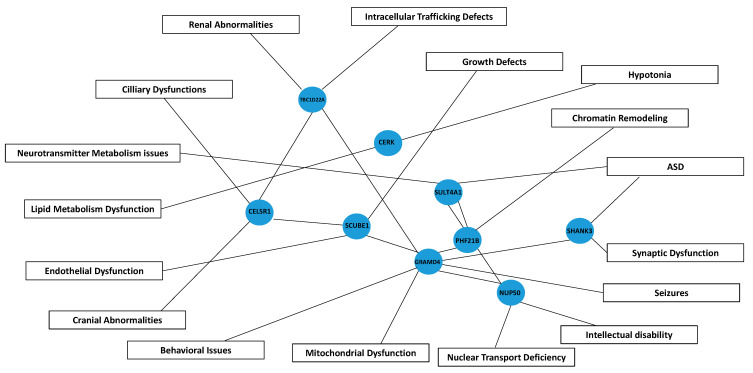
Schematic pathway diagram illustrating the interactions between genes and their associated contributions to phenotypic manifestations in Phelan–McDermid syndrome. Blue nodes represent genes (e.g., *SHANK3*, *GRAMD4*, *CELSR1*), while boxes denote phenotypes and functional disruptions, including seizures, cranial abnormalities, and lipid metabolism dysfunctions, among others. Connecting lines indicate putative interactions/connections, illustrating how gene disruptions may lead to specific clinical outcomes in PMS. ASD; Autism spectrum disorder.

The principal functions, metabolic pathways, and biological processes associated with the genes highly susceptible to haploinsufficiency in Regions R1 to R3 are summarized schematically in Figure 8.

## 3. Discussion

This retrospective study tests the hypothesis that genes beyond *SHANK3* contribute to specific clinical features of Phelan–McDermid syndrome (PMS). The findings reinforce the concept of PMS as a contiguous gene syndrome, aligning with recent work by [19], while challenging the notion that *SHANK3* alone accounts for the disorder’s phenotypic diversity. Although *SHANK3* haploinsufficiency remains a central pathogenic mechanism, additional genes within the 22q13.31–22q13.33 region appear to influence key PMS features.

### 3.1. SHANK3 as the Core Driver, but Not the Sole Contributor

*SHANK3* plays a pivotal role in synaptic development and function, explaining its association with hallmark neurological and behavioral traits of PMS, such as intellectual disability, ASD, and hypotonia. However, interstitial deletions that exclude *SHANK3* but still present with PMS-like features suggest the involvement of additional genes, particularly in cases involving deletions larger than 0.25 Mb.

This study systematically defines three critical haploinsufficient regions (R1–R3) within 22q13.31–q13.33, emphasizing Region 2 (hg38, chr22: 44,702,204–47,175,693) as consistently associated with functional impairments reflected in GFAP scores. Interestingly, several genes within Region 2, including *CERK*, *TBC1D22A*, *CELSR1*, and *GRAMD4* were found to be implicated in seizures, renal abnormalities, lymphedema, macrocephaly, and developmental delay in PMS [8,16,20,21,22].

Finally, we also highlight that the mean haploinsufficiency score of genes in Region 1 (which includes *SHANK3*) matches that of Region 2, it could suggest a comparable putative contribution to PMS pathogenesis of Region 2.

### 3.2. Deletion Size as a Key Determinant

GFAP analyses confirm that phenotypic severity correlates with deletion size, reinforcing the idea that additional genes modulate the clinical spectrum of PMS. Larger deletions are associated with poorer functional outcomes [3,23,24], whereas smaller deletions show clinical profiles similar to those observed in individuals with *SHANK3* SNVs [3,4]. Even interstitial deletions not involving *SHANK3* may present with PMS-like traits [7], supporting their inclusion in future PMS research.

### 3.3. Clusters and Phenotypic Variability

Phenotypic heterogeneity was evident across deletion size-based clusters. Cluster 3 (small deletions) exhibited milder phenotypes, while Cluster 4 (large deletions) was associated with more severe impairments, consistent with a cumulative effect of gene loss. Interestingly, the *SHANK3* SNV (“Cluster 5”) showed functional overlap with Cluster 3, particularly in speech and motor outcomes, reinforcing the functional importance of *SHANK3*. Altogether also support the participation of genes beyond *SHANK3*.

### 3.4. Specific Phenotypes and Genetic Correlations

This study refines genotype–phenotype correlations for seizures, renal anomalies, and lymphedema, particularly in relation to deletions involving *CELSR1* and *GRAMD4* [22,25]. Larger deletions also strongly correlate with hypotonia and expressive language impairments [3]. Interestingly, the prevalence of ASD remained constant across deletion sizes, supporting *SHANK3*’s central role in this phenotype [2]. Conflicting findings regarding deletion size and ASD [19,23] may reflect diagnostic challenges in more severely affected individuals, where profound impairments may mask features of ASD or ADHD. Deletion size also influences cranial morphology (macrocephaly) associated with deletions > 3.5 Mb and microcephaly with smaller deletions [8,21]. A threshold of 2.7 Mb was associated with more severe developmental impairments, in agreement with prior breakpoint mapping studies [24].

### 3.5. Implications of Additional Genes

Candidate genes in Region-2 (including *CERK*, *TBC1D22A*, *CELSR1*, and *GRAMD4*) are likely contributors to PMS pathophysiology. *CERK* (ceramide kinase) is involved in lipid metabolism and potentially related to hypotonia and immune dysregulation. *TBC1D22A* participates in intracellular trafficking, associated with seizures and renal anomalies. *CELSR1* is implicated in craniofacial and renal abnormalities, consistent with its role in planar cell polarity. *GRAMD4* regulates apoptosis and is linked to seizures, lymphedema, and sensory processing dysfunction. These genes play crucial roles in the regulation of cell signaling, cytoskeleton organization, apoptosis, and immune response, participating in biological pathways essential for the maintenance of cellular homeostasis and embryonic development. Indeed, a few interactions among these genes (*CERK*, *TBC1D22A*, *CELSR1*, *GRAMD4)* have been identified in the available protein interaction databases. Interactions between CERK and TBC1D22A proteins have been reported in HeLa cells, detected by affinity capture and mass spectrometry techniques (BioGRID; http//:www.thebiogrid.org (accessed on 17 February 2025)). In addition, GRAMD4 is predicted to interact with other proteins, such as TBC1D22A and RABL2B (https://string-db.org/ (accessed on 16 February 2025)). Those interactions were based on evidence of gene co-expressions (with medium confidence), which suggests that both proteins may be involved in related cellular processes (Figure 7A). In the case of RABL2B interactions, these were also experimentally established (https://string-db.org/ (accessed on 16 February 2025)).

Regarding some functionality of these genes (*TBC1D22A*, *CELSR1*, and *GRAMD4)*, we propose previously a region of 4.5 to 8 Mb from the telomere, strongly associated with macrocephaly in PMS [3], also supported by this study, and aligned with other works [21], suggesting to *GRAMD4* associated with macrocephaly. *TBC1D22A* has been associated with structural or functional abnormalities of the head or the central nervous system (CNS), such as seizures, schizophrenia, or bipolar disorder [26,27]. In addition, *CELSR1*-deficient mouse has found to have microcephaly and cortical hypoplasia [28], but also with defects in neural tube and caudal agenesis [29], lymphedema [17], or several kidney disorders [22].

Additional genes from Regions 2 and 3 (*PHF21B*, *SULT4A1*, *SCUBE1*, and *NUP50*) we speculate that they may further contribute to cognitive deficits, neurodevelopmental features, and growth abnormalities.

### 3.6. Genetic Interactions and Future Directions

We hypothesize that interactions among these candidate genes and *SHANK3* may account for the broad phenotypic variability observed in PMS. Currently, no direct interactions between the *CERK*, *TBC1D22A*, *CELSR1*, *GRAMD4*, and *SHANK3* genes have been identified in the available protein interaction databases. Although their roles in neuronal development, cell signaling, and synaptic organization suggest that they could be involved in broader interaction networks that affect neuronal function, behavior, and beyond. On the other hand, we do know that *SHANK3* is predicted to interact with at least six other genes located in 22q13.3—*PLXNB2*, *BRD1*, *CELSR1*, *PHF21B*, *SULT4A1*, and *TFC20*—underscoring the genetic complexity of the syndrome [20]. In this sense, in Figure 8 we describe a schematic pathway diagram illustrating the interactions between genes and their associated contributions to phenotypic manifestations in Phelan–McDermid syndrome.

In summary, our findings confirm significant functional variation among PMS patients based on deletion size, further supporting the classification of PMS as a contiguous gene syndrome. Future research should investigate whether these genes modulate *SHANK3* expression or function, potentially revealing novel therapeutic targets.

## 4. Materials and Methods

### 4.1. PMS Spanish-Based Registry

This registry comprises 245 patients previously recruited through INGEMM (Institute of Medical and Molecular Genetics, Hospital Universitario La Paz, Madrid), in collaboration with the Spanish Association of PMS and the Argentine PMS Group [3]. Clinical data were obtained via two structured questionnaires completed by physicians, clinical geneticists, and caregivers, following informed consent. The study was approved by the Ethics Committee of Hospital Universitario La Paz (PI: 2735 HULP, Madrid, Spain). Parents or guardians provided informed consent.

In addition to clinical records, the registry includes curated genetic data derived from MLPA, karyotyping, FISH, and chromosomal microarray analysis (including high-resolution CMA in some cases). Cognitive behavioral evaluations and quality-of-life assessments were also conducted in a subset of 30 individuals. For this study, only data from the two primary questionnaires were analyzed, and hierarchical criteria were applied (e.g., nephro-urogenital anomalies as an umbrella category).

#### Generation of the Database from the “Spanish PMS Registry”

A comprehensive database was constructed using SPSS (version 29.0.2), with patient data pseudo-anonymized and coded from 1 to 213. The database contains both continuous and categorical variables. Key continuous variables include age at diagnosis, deletion size, GFAP score, number of deleted genes, and number of OMIM-annotated genes (with corresponding inheritance types, based on OMIM, updated as of 21 November 2024). Haploinsufficiency scores were also assigned. Sixty-four categorical variables representing various phenotypic features were incorporated.

The cohort includes individuals with confirmed diagnosis of PMS (mostly not related) from across Spain (*n* = 181) and South America (*n* = 32), and they were recruited between years 2008–2022. Twenty-seven of these individuals had incomplete clinical or molecular data and were not included in this study, but they form part of the registry. All cases analyzed are deposited in the DECIPHER Genomics repository under accession numbers 432868–433079 (IMMGPMS1–IMMGPMS211). The female-to-male ratio was 1.108:1 (112/101), with ages ranging from birth to 62 years. The majority of individuals with PMS in our cohort are of pediatric age (between 0 and 16 years old, 149 patients; 69.95%). The mean age at diagnosis was around 6 years old for deletions and around 8 years for the group with sequence variants in *SHANK3*. The mean age at evaluation were around 12.5 years and 11 years for deletions and *SHANK3* sequence variants, respectively. Full demographic and complete clinical descriptions are available in [3].

### 4.2. The Study

This retrospective, experimental study was conducted using data from the Spanish PMS Registry, processed in SPSS (v. 29.0.2). Inclusion criteria were based primarily on the availability of high-resolution microarray genetic data. Chromosomal coordinates were standardized to the latest genome build, GRCh38/hg38, using the UCSC LiftOver tool (https://genome.ucsc.edu/ (accessed on 15 January 2025)).

### 4.3. Genetic Analysis

Of the 213 individuals in the cohort, 192 (90.14%) had deletions in the 22q13.3 region, while the remaining 21 patients (9.86%) harbored pathogenic or likely pathogenic single nucleotide variants (SNVs) in the *SHANK3* gene. Deletion sizes ranged from ~0.01 Mb to over 10 Mb. Among those with deletions, 176 (91.66%) had terminal deletions, of which 13 (7.38%) were associated with translocations (de novo or inherited) and 20 (11.36%) with ring chromosome 22. Interstitial deletions including *SHANK3* were identified in 13 individuals (6.77%), with an additional three interstitial deletions not involving *SHANK3* (1.57%) [3]. Postzygotic mosaicism was identified in 17 cases (9.98%), and germline mosaicism was suspected in two others (0.94%).

The mean deletion size was 3.52 ± 2.54 Mb. Based on deletion size, individuals were classified into two groups: small deletions (≤0.25 Mb; 21.7%) and large deletions (>0.25 Mb; 78.3%). The 0.25 Mb threshold was selected because *SHANK3* is located approximately 0.1–0.2 Mb from the telomere. The SNV group included pathogenic or likely pathogenic variants: 17 frameshift, one splice-site, one nonsense, and two missense variants of uncertain significance/likely pathogenic [3].

### 4.4. Patients’ Global Functional Assessment (GFAP) Construct

The GFAP (Global Functional Assessment of the Patients) test is a scoring system developed to quantify the overall clinical severity of individuals with Phelan–McDermid syndrome. It was designed using weighted clinical features from the Human Phenotype Ontology (HPO), based on their observed frequency in the studied cohort. Clinical traits occurring in more than 70% of patients were scored 10 points, those between 35 and 70% received 5 points, 20–35% achieved 2 points, and those under 20% were scored 1 point. The total GFAP score thus reflects the accumulation of core clinical manifestations, including developmental delay, speech impairment, hypotonia, and behavior anomalies. Its validity was confirmed through principal component analysis, ensuring that the score accurately reflects severity across diverse genetic subtypes. This allows comparisons among patients and subgroups, revealing, for instance, that larger deletions tend to be associated with higher GFAP scores, i.e., greater severity (for more information see [3]).

### 4.5. Global Haploinsufficiency Score of Deleted Genes in 22q13.31–q13.33

To identify regions of potential haploinsufficiency within 22q13.31–q13.33 (8.3 Mb), a composite score was calculated using five published haploinsufficiency prediction algorithms. For each gene, the following criteria were applied: (a) pLI [30]: <0.1 = 0 pts; 0.11–0.89 = 2 pts; >0.9 = 15 pts; (b) LOEUF [31]: <0.268 = 15 pts; >0.268 = 1 pt; (c) sHet [32]: >0.1 = 15 pts; <0.1 = 1 pt; (d) pHaplo [31]: >0.86 = 15 pts; 0.56–0.85 = 10 pts; <0.55 = 0 pts; (e) pTriplo [31]: >0.94 = 15 pts; 0.69–0.93 = 10 pts; <0.68 = 0 pts. These scores were summed to generate a global haploinsufficiency index for each gene.

### 4.6. Statistical Analysis

Statistical analyses were performed using SPSS Statistics v.29.0.2 (IBM Corporation, Chicago, IL, USA). Both univariate and multivariate methods were employed. Descriptive statistics (means, standard deviations) were calculated for continuous variables, while frequency distributions were used for categorical variables (coded as “1” for presence and “0” for absence of a feature).

Inferential statistics included Pearson and Spearman correlation coefficients, Chi-square tests, and ANOVA with Bonferroni and T3 Dunnett post hoc tests. Unsupervised clustering (Ward’s method) was applied to group individuals based on deletion size and/or GFAP score, facilitating comparative phenotypic analyses. Logistic and linear regression models were used to evaluate the predictive value of genetic variables for specific clinical outcomes. Statistical significance was set at *p* < 0.05, with *p* < 0.01 considered highly significant.

### 4.7. Study Limitations

While this study provides substantial evidence for genotype–phenotype correlations in PMS, several limitations should be acknowledged. (a) Sample Size: The relatively small number of patients with interstitial deletions limits the generalizability of findings related to genes outside SHANK3. (b) Lack of Longitudinal Data: As the data are cross-sectional, they do not capture changes in phenotypes over time. Longitudinal follow-up is needed to assess developmental trajectories and progression. (c) Non-Genetic Influences: The potential roles of epigenetic and environmental factors in shaping phenotypes remain underexplored. Integrating multi-omics data (e.g., transcriptomics, proteomics) could help elucidate pathways mediating gene dosage effects.

## 5. Conclusions

The findings of this study have important implications for the diagnosis and management of Phelan–McDermid syndrome (PMS), particularly in the context of comprehensive genomic profiling. Routine diagnostic workflows should incorporate high-resolution genomic technologies to accurately delineate deletion breakpoints and identify genes affected beyond *SHANK3*.

Future Personalized Interventions: Understanding the impact of deletion size and the contribution of specific gene losses may inform personalized therapeutic strategies. For example, individuals with large deletions may benefit from earlier and more intensive interventions focused on speech and motor development.

Prognostic Value of GFAP Scores: The GFAP score provides a quantitative, functional assessment of clinical severity and may serve as a valuable prognostic tool to prioritize therapeutic planning.

In conclusion, this study reaffirms the central role of *SHANK3* in PMS while underscoring the relevance of additional genes in contributing to the disorder’s phenotypic complexity. By characterizing PMS as a contiguous gene syndrome, these findings support the implementation of more precise diagnostic practices and the development of tailored, gene-informed interventions.

## Figures and Tables

**Table 1:** Pearson’s correlations between continuous variables.

Variable-1	Variable-2	Pearson’s r	*p*-Value (Bilateral)	Interpretation
Deletion size	GFAP * score	0.359	<0.0001	Positive correlation: larger deletions linked to higher GFAP scores.
	Number of genes	0.949	<0.0001	Strong positive correlation.
	Number of OMIM genes	0.894	<0.0001	Strong positive correlation.
	Number of OMIM genes with AD	0.844	<0.0001	Strong positive correlation.
	Number of OMIM genes with AR	0.819	<0.0001	Strong positive correlation.
GFAP score	Number of genes	0.377	<0.0001	Positive correlation: more genes lost lead to higher GFAP scores.
	Number of OMIM genes	0.378	<0.001	Strong positive correlation.
	Number of OMIM genes with AD	0.280	<0.001	Strong positive correlation.
	Number of OMIM genes with AR	0.376	<0.001	Strong positive correlation.
Age at diagnosis	Deletion size	−0.249	0.001	Inverse correlation: larger deletions diagnosed earlier.
	GFAP score	−0.183	0.009	Inverse correlation: higher GFAP scores diagnosed earlier.

* GFAP, global functional assessment of the patient; OMIM, online mendelian inheritance in man; AD, autosomal dominant inheritance; AR, autosomal recessive inheritance.

**Table 2:** Relationship between categorical variables and genetic clusters, showing statistically significant associations as determined by the Chi-square test.

Chi-Square Test Among Clusters	Clusters with Statistical Associations	Value	df	Significance (*p*) (Bilateral)
Ability to make Sentences	CL1&3; CL2&3; CL3&4	35.598	4	<0.0001 **
Walk independent before 15 months	CL1&3; CL2&3; CL3&4	42.889	4	<0.0001 **
Alteration of Growth PC > 95% or PC < 3%	CL4&3; CL4&5	18.041	1	0.021 *
Hypotonia	CL1&3; CL2&3; CL3&4	25.204	4	<0.0001 **
Deep set eyes	CL2&5	11.764	4	0.02 *
Full brow	CL1&5; CL2&5	11.764	4	0.019 *
Full/puffy eyelids	CL1&3; CL2&3	11.764	4	0.004 *
Full/puffy cheeks	CL1&3	10.342	4	0.035 *
Toe syndactyly	CL3&4	21.489	4	<0.0001 **
Large and fleshly hands	CL2&3	14.572	4	0.006 *
Sphincter control	CL1&2; CL1&3; CL1&5; CL2&5; CL4&5	29.789	4	<0.0001 **
Nephro-urological anomalies	CL1&4; CL3&4; CL4&5	16.509	4	0.002
Obesity	No cases in CL3, CL4, CL5	10.410	4	0.034 ^FeT^ *
Lymphedema	CL1&2; CL1&4; no cases in CL5	22.055	4	<0.0001 **
High sensitive (touch, caress, skim)	CL3&5	11.133	1	0.025 *

V: test statistic value; gl: degrees of freedom; *p*: *p*-value. The table presents only those variables demonstrating significant differences across clusters, highlighting their interrelation. CL, Cluster; FeT, Fisher’s exact Test. ** A *p*-value less than 0.001 on Student’s *t*-test indicates a highly significant difference, while a *p*-value less than 0.05 * indicates a significant difference.

**Table 3:** Relationship between categorical variables and genetic groups defined by deletion size and *SHANK3* variants, showing statistically significant associations as determined by the Chi-square test.

Chi-Square Test	Group 1	Group 3				
(a)	Deletions <0.25 Mb (N = 37)	*SHANK3* SNVs (N = 18)	Value	df	Significance (*p*) (Bilateral)	Interpretation
Ability to make sentences	24	5	4.102	1	0.038 *	Group 3 has a significantly lower chance of having the ability to make sentences compared to Group 1
Teeth anomalies	16	2	5.82	1	0.028 ^FeT^ *	Group 3 has a significantly lower chance of having teeth anomalies compared to Group 1
Sphincter control	8	14	4.71	1	0.030 *	Group 3 has a significantly higher chance of having sphincter control compared to Group 1
Sleeping problems	7	9	5.83	1	0.016 *	Group 3 has a significantly higher chance of having sleeping anomalies compared to Group 1
Highly sensitive (touch, caress, skim)	11	12	7.11	1	0.008 *	Group 3 has a significantly higher chance of being very sensitive to touch compared to Group 1
Chi-square test	Group 2	Group 3				
(b)	Deletions >0.25 Mb (N = 150)	*SHANK3* SNVs (N = 18)	Value	df	Significance (*p*) (bilateral)	
Walk independently before 15 months	30	13	8.843	1	0.004 ^FeT^ *	Group 3 (SNVs) have a much higher chance of walking before 15 months compared to Group 2
Alteration of Growth PC > 95% or PC < 3%	75	2	25.469	1	<0.0001 ^FeT^ *	Group 3 is significantly less likely to exhibit growth anomalies compared to Group 2
Hypotonia	121	10	4.308	1	0.038 *	Group 3 has a significantly lower chance of having hypotonia compared to Group 2
Epicanthal folds	43	1	4.034	1	0.045 ^FeT^ *	Group 2 has a significantly higher chance of having epicanthus compared to Group 3
Deep set eyes	38	0	5.575	1	0.014 ^FeT^ *	Group 3 has no chance of having deep set eyes compared to Group 2
Full brow	64	1	8.691	1	0.003 ^FeT^ *	Group 3 is significantly less likely to exhibit full brown compared to Group 2
Chi-square test	Group 1	Group 2				
(c)	Deletions <0.25 Mb (N = 37)	Deletions >0.25 Mb (N = 150)	Value	df	Significance (*p*) (bilateral)	Interpretation
Ability to make Sentences	24	22	33.095	1	<0.0001 *	Group 2 has a very significantly lower chance of having the ability to make sentences compared to Group 1
Walk independently before 15 months	19	30	15.086	1	<0.0001 *	Group 1 has a much higher chance of walking before 15 months compared to Group 2
Alteration of Growth PC > 95% or PC < 3%	9	75	7.723	1	<0.005 *	Group 1 is significantly less likely to exhibit growth anomalies compared to Group 2
Hypotonia	21	121	9.285	1	0.002 *	Group 2 has a significantly higher chance of having hypotonia compared to Group 1
Strabismus	4	45	5.652	1	0.021 ^FeT^ *	Group 2 has a very higher chance of having strabismus compared to Group 1
Full/puffy eyelids	2	41	8.059	1	0.004 ^FeT^ *	Group 2 has a very higher chance of having full/puffy eyelids compared to Group 1
Full/puffy cheeks	3	39	5.455	1	0.026 ^FeT^ *	Group 2 has a very higher chance of having full/puffy cheeks compared to Group 1
Toe syndactyly	3	48	8.541	1	0.003 ^FeT^ *	Group 2 has a very higher chance of having toe syndactyly compared to Group 1
Large/fleshly hands	11	90	15.948		0.001 *	Group 2 has a very higher chance of having large/fleshly hands compared to Group 1
Sphincter control	9	17	4.184	1	0.041 *	Group 1 has a significantly higher chance of having sphincter control compared to Group 2
Pulling the hair (to others or themself)	3	39	5.455	1	0.026 ^FeT^ *	Group 2 has a significantly higher chance of having pulling the hair compared to Group 1

V: test statistic value; gl: degrees of freedom; *p*: *p*-value. The table displays only those variables with significant differences, emphasizing their interrelations across genetic subgroups. FeT, Fisher’s exact test. * *p*-value less than 0.05 indicates a significant difference.

**Table 4:** Statistical analysis of GFAP scores in individuals with deletions (N = 189), comparing those who present the clinical feature (coded as “1”) versus those who do not (coded as “0”).

Descriptive and Frequencies Statistics						
		Median Mb	Median GFAP	Mean GFAP	Frequency	Percentage
Gender	Male	-	-	-	85	44.97%
	Female	-	-	-	104	55.02%
	Total	-	-	-	189	100%
^&^ Developmental delay (walk before/after 15 months)	>15 months	3.47	112	110.10 ± 38.26 **	138	73.79%
	Normal	-	-	-	-	-
	<15 months	1.39	72	70.26 ± 38.99	49	26.20%
	Total	-	-	-	187	100%
Ability to make sentences	Not	3.90	136	132.21 ± 30.99	143	76.47%
	Yes	0,28	65	60.56 ± 37.78 **	44	23.52%
	Total	-	-	-	187	100%
Hypotonia	Not	0,77	52	53.66 ± 34,31	45	24.06%
	Yes	30.85	115.5	114.23 ± 33.02 **	142	75.93%
	Total	-	-	-	187	100%
Seizures	Not	2.88	94.34	94.55 ± 41.89	129	68.98%
	Yes	4.14	115.5	111.03 ± 40.91 *	58	31.01%
	Total	-	-	-	187	100%
^$^ Craneal anomalies	Normal	3.03	107	100.58 ± 44.03	102	54.25%
	Microcephaly	2.55	95	90.05 ± 38.70	37	19.68%
	Macrocephaly	5	107	106.63 ± 41.27	49	26.06%
	Total	-	-	-	188	100%
Syndactily	Not	2.85	97	94.34 ± 43.06	136	72.72%
	Yes	4.17	115	113.84 ± 36.47 **	51	27.27%
	Total	-	-	-	187	100%
Ophtalmological alterations	Not	2.91	97	93.39 ± 40.19	146	78.07%
	Yes	3.94	126	121.97 ± 41.99 **	41	21.92%
	Total	-	-	-	187	100%
Nephro-urological alterations	Not	2.95	101	95.49 ± 40.61	146	77.65%
	Yes	4.25	122	116.07 ± 45.34 **	42	22.34%
	Total		-	-	188	100%
Dermatological anomalies	Not	2.95	101	96.52 ± 42.58	146	77.65%
	Yes	4.07	112.5	112.5 ± 40.15 *	42	22.34%
	Total	-	-	-	188	100%
Obesity	Not	3.16	102	99.92 ± 42.70	184	97.87%
	Yes	6.91	112.5	107.5 ± 33.27	4	2.12%
	Total	-	-	-	188	100%
Lymphedema	Not	2.94	101	97.23 ± 42.50	169	90.37%
	Yes	7.35	125.5	122.5 ± 31.69 **	18	962%
	Total	-	-	-	187	100%

The table includes only variables that showed statistically significant differences, highlighting associations between specific clinical traits and functional impairment as measured by GFAP. ** A *p*-value less than 0.001 on Student’s *t*-test indicates a highly significant difference, while a *p*-value less than 0.05 * indicates a significant difference. ^&^ (F: 12.609; mean square: 97.792; df < 1; *p* < 0.001); ^$^ (F: 7.634; mean squared: 58.520; df: 2; *p* < 0.001).

**Table 5:** Gene clustering with a score > 30. Highly haploinsufficient genes. Genomic coordinates in GCRh38 (hg38).

GEN	COORDINATES	SCORE (AU)
*SCUBE1*	chr22: 43197280–43343372	32
*SULT4A1*	chr22: 43824509–43862513	50
*PHF21B*	chr22: 44881162–45010005	46
*NUP50*	chr22: 45163925–45188017	34
*FBLN1*	chr22: 45502238–45601135	46
*CELSR1*	chr22: 46360834–46537620	56
*GRAMD4*	chr22: 46576012–46679790	70
*TBC1D22A*	chr22: 46762617–47175699	34
*BRD1*	chr22: 49773283–49827873	66
*PIM3*	chr22: 49960768–49964072	55
*PLXNB2*	chr22: 50274979–50307646	32
*SBF1*	chr22: 50443219–50483923	32
*MAPK8IP2*	chr22: 50600793–50613981	60
*SHANK3*	chr22: 50674408–50733212	45
		Average *

* The average score value in genes >30 is 47. AU, arbitrary units.

**Table 6:** Gene clustering with GFAP score > 20. Moderately haploinsufficient genes. Genomic coordinates in GCRh38 (hg38).

GEN	COORDINATES	SCORE (AU)
*TTLL12*	chr22: 43197280–43187134	22
*MPPED1*	chr22: 43411196–44507848	26
*PRR5*	chr22: 44668547–44737681	28
*WNT7B*	chr22: 45920366–45977162	27
*PPARA*	chr22: 46150521–46243755	27
*ZBED4*	chr22: 49853844–49890080	28
*ALG12*	chr22: 49900229–49918438	27
*RABL2B*	chr22: 50767501–50783667	26
		Average *

* The mean value of the score in genes > 20 is 26.375. AU, arbitrary units.

## Data Availability

Additional supporting information is available in the Supplementary Materials section online. All patient data have been submitted to the DECIPHER database (http://www.deciphergenomics.org (accessed on 12 April 2022)).

## Supplementary Materials

The following supporting information can be accessed online at: https://www.mdpi.com/article/10.3390/ijms26104653/s1.
