# Supplementary material for: Genotype–Phenotype Associations in Phelan–McDermid Syndrome: Insights into Novel Genes Beyond SHANK3
Source: Int J Mol Sci. 2025 May 13;26(10):4653. doi: 10.3390/ijms26104653 (PMC12111097; doi:10.3390/ijms26104653)
Supplement: Supplementary file 1 [file ijms-26-04653-s001.zip › newSupplemental data - copia.pdf]

International Journal of *Molecular Sciences*

MDPI

Supplemental data.

2.4. Exploring a candidate gene analysis. GFAP analyses of individuals showing versus not showing a condition for categorical variables. Continued-

2.4.3. Seizures

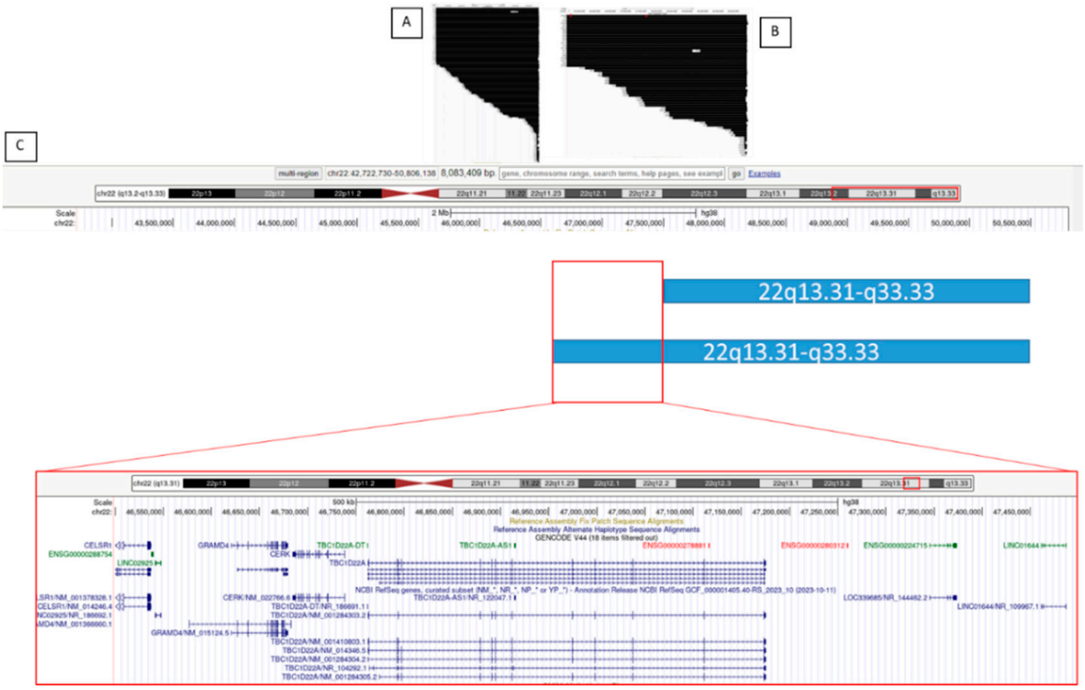

Figura S1 Supplemental data. Graphical representation in Genome Browser of patients with seizures vs. not. A; patients without seizures, B; patients with seizures, C; differences in the haploinsufficiency region between groups A and B. Genomic coordinates in GCRh38 (hg38).

We found that the genes that mainly differ between the two conditions are: *TBC1D22A, CELSR1, CERK y GRAMD4*.

2.4.4. Hypotonia

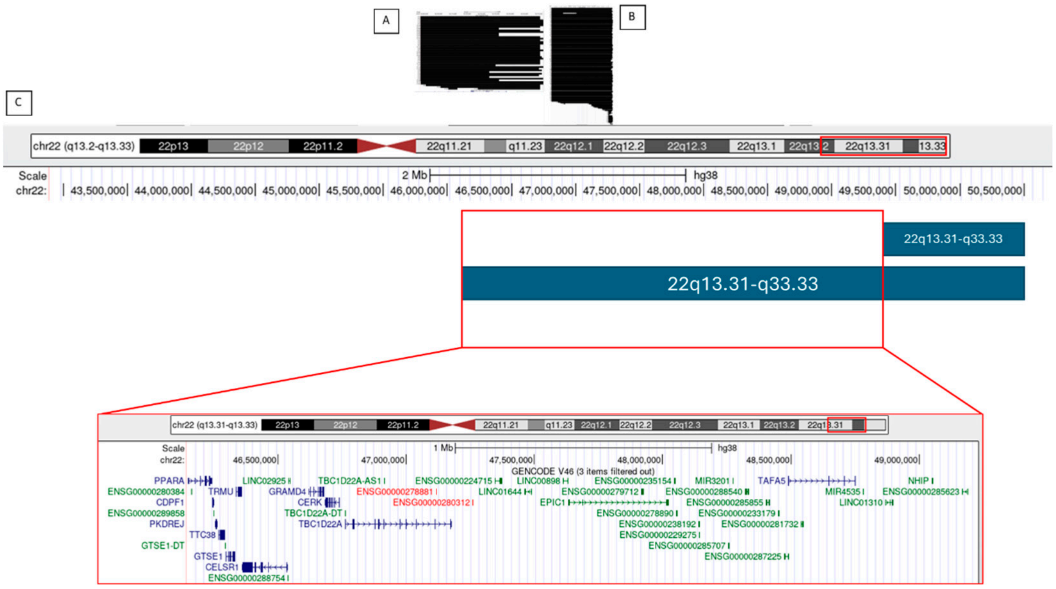

Figure S2. Supplemental data. Graphical representation in Genome Browser of patients with hypotonia vs. not. A; patients without obesity, B; patients with obesity, C; haploinsufficiency region. Genomic coordinates in GRCh38 (hg38).

We found that the genes that differ between the two conditions are: *PPARA, CDPF1, PKDREJ, TTC38, TRMU, GTSE1, CELSR1, TBC1D22A, CERK, GRAMD4, EPIC1, TAFA5, MIR4535 1, NPHP1*

2.4.5. Opthtalmological anomalies

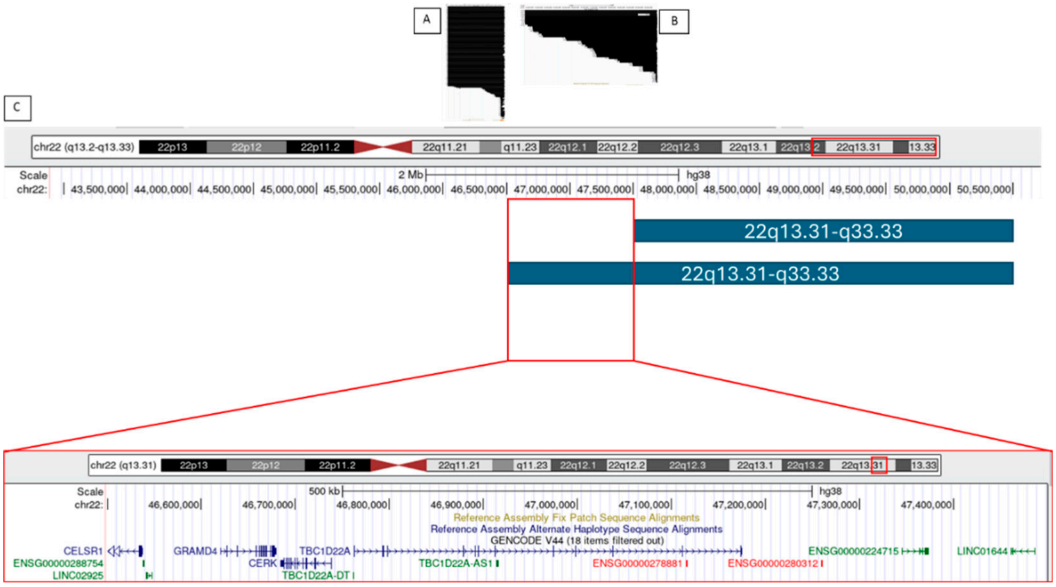

Figure S3. Supplemental data. Graphical representation in Genome Browser of patients with opthalmological anomalies vs. not. A; patients without opthalmological anomalies, B; patients with opthalmological anomalies, C; haploinsufficiency region. Genomic coordinates in GRCh38 (hg38). E We found that the genes that mainly differ between the two conditions are: *TBC1D22A, CERK, GRAMD4 Y CELSR1*.

2.4.6. Dermatological anomalies

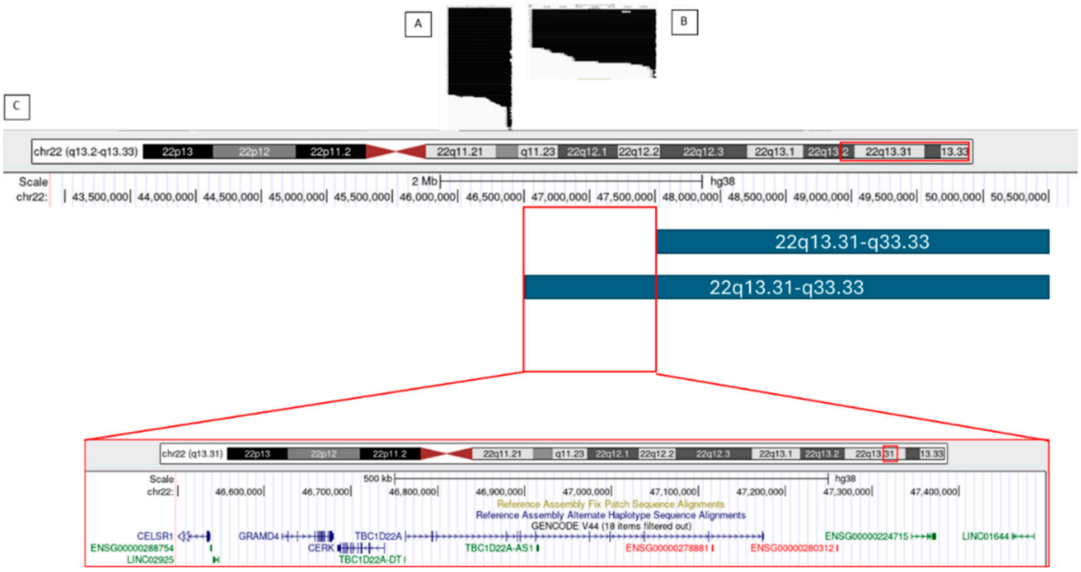

Figure S4. Supplemental data. Graphical representation in Genome Browser of patients with Dermatological anomalies vs. not. A; patients without Dermatological anomalies, B; patients with Dermatological anomalies, C; haploinsufficiency region. Genomic coordinates in GRCh38 (hg38). We found that the genes that mainly differ between the two conditions are: *TBC1D22A, CERK, GRAMD4* y *CELSR1*.

2.4.7. Syndactily

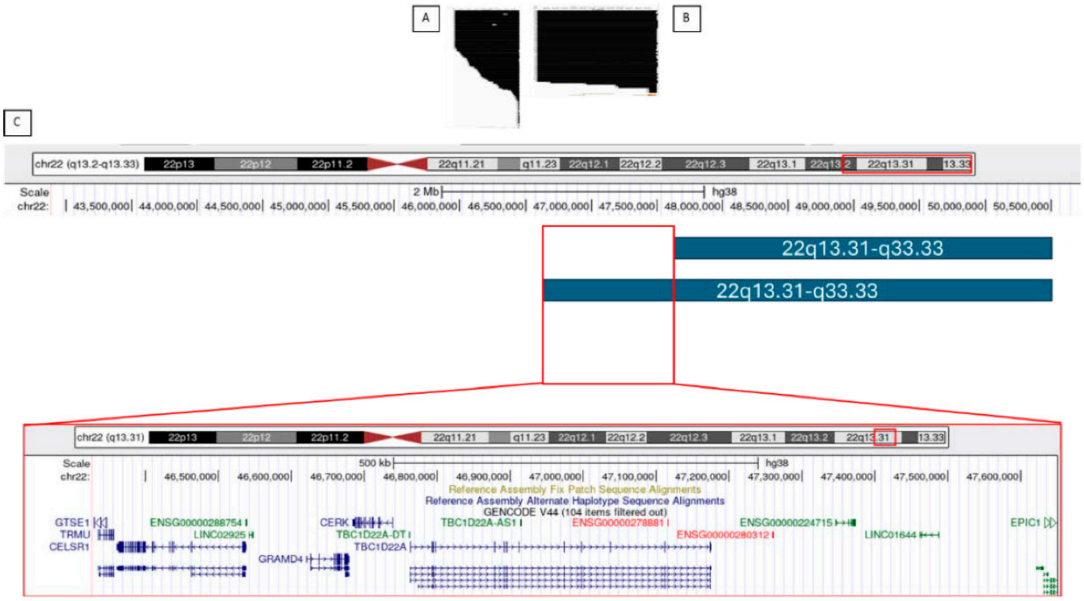

Figure S5. Supplemental data. Graphical representation in Genome Browser of patients with Syndactily vs. not. A; patients without Syndactily, B; patients with Syndactily, C; haploinsufficiency region. Genomic coordinates in GRCh38 (hg38).

We found that the genes that mainly differ between the two conditions are: *TBC1D22A, CERK, GRAMD4, CELSR1, TRMU* y *GTSE1*.

2.4.8. Craneal size anomalies

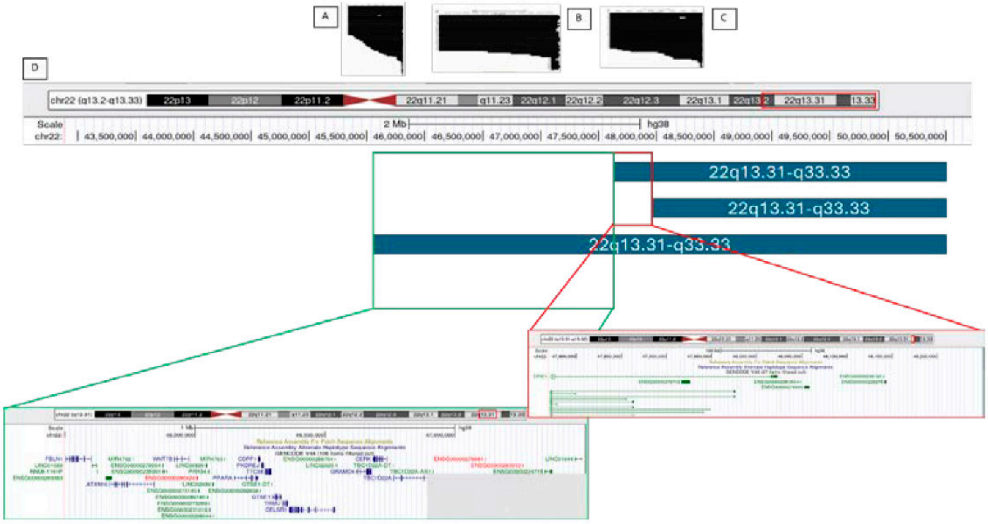

Figure S6. Supplemental data. Graphical representation in Genome Browser of patients with cranial size anomalies vs. not. A; patients with microcephaly, B; patients with

macrocepahaly, C; patients without anomalies, D; haploinsufficiency region. Genomic coordinates in GRCh38 (hg38).

We found that the genes that mainly differ between the two conditions are: *TBC1D22A, CERK, GRAMD4, CELSR1, TRMU, GTSE1, PPARA, TTC38, PKDREJ, CDPF1, WNT7B, ATXN* y *FBLN1*.

2.4.9. Ability make sentences

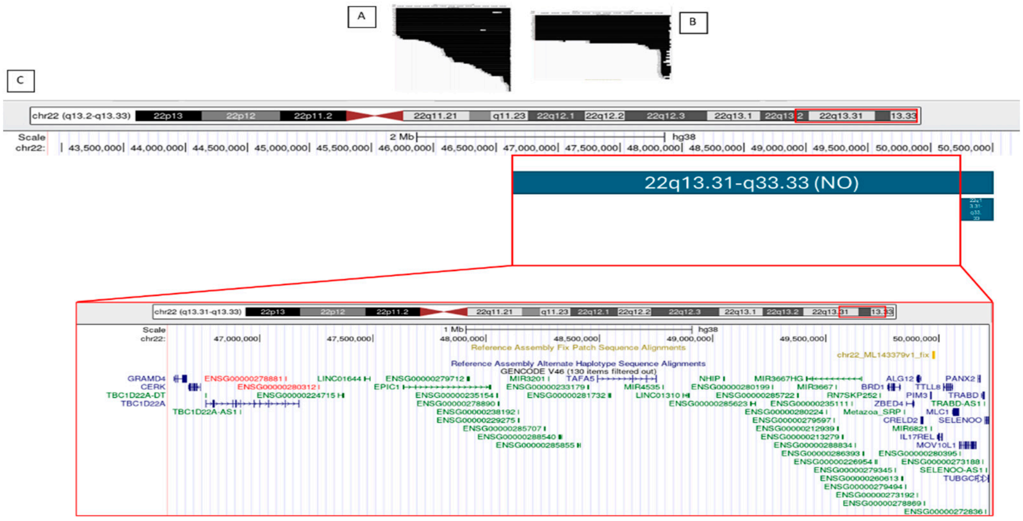

Figure S7. Supplemental data. Graphical representation in Genome Browser of patients with ability to make sentences vs. not. A; patients with abilities, B; patients without abilities, C; haploinsufficiency region. Genomic coordinates in GRCh38 (hg38).

We found that the genes that mainly differ between the two conditions are: *TBC1D22A, CERK, GRAMD4, TAFA5, ALG12, PANX2, BRD1, TTLL8, PIM3, TRABD, ZBED4, MLC1, CRELD2, SELENOO, IL17REL, MOV10L1* y *TUBGCP6.*

2.4.10. Developmental delay (to walk before 15 months)

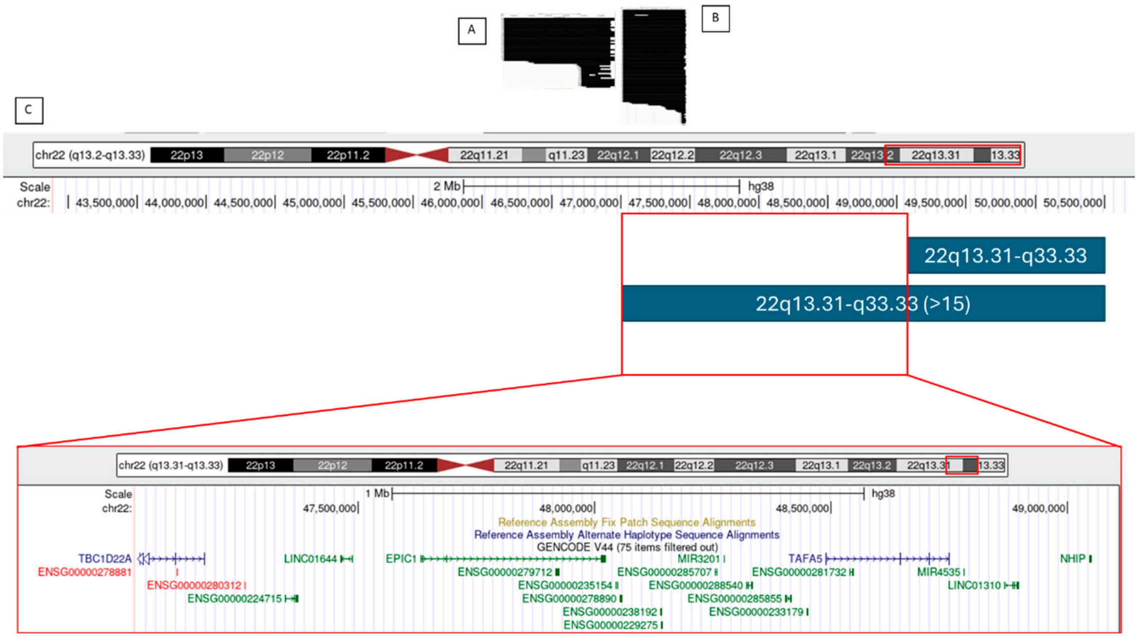

Figure S8. Supplemental data. Graphical representation in Genome Browser of patients with ability to walk before 15 months vs. not. A; patients without abilities, B; patients with abilities, C; haploinsufficiency region. Genomic coordinates in GRCh38 (hg38).

We found that the genes that mainly differ between the two conditions are: son: *TBC1D22A* y *TAFA5*.

2.4.11. Obesity

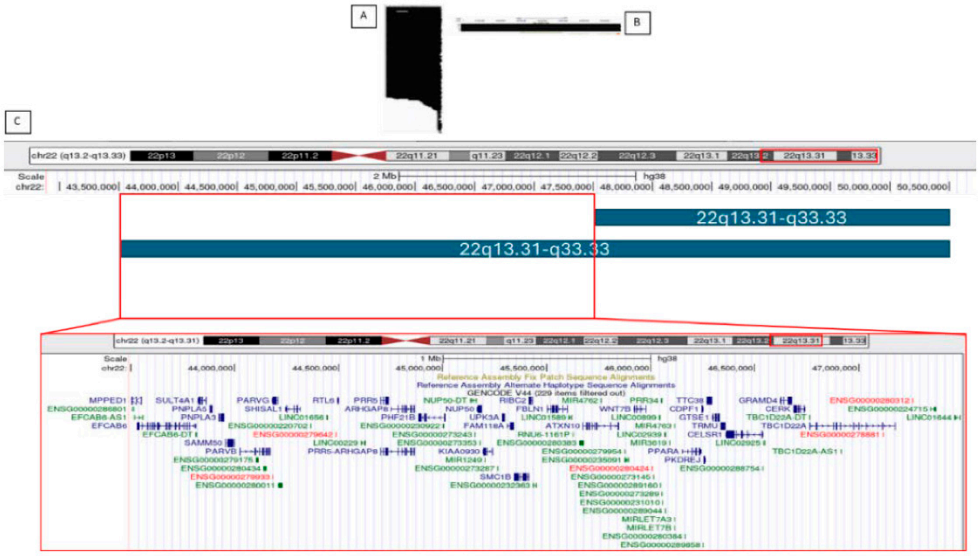

Figure S9. Supplemental data. Graphical representation in Genome Browser of patients with obesity vs. not. A; patients without obesity, B; patients without obesity, C; haploinsufficiency region. Genomic coordinates in GRCh38 (hg38).

We found that the genes that mainly differ between the two conditions are: *TBC1D22A, CERK, GRAMD4, CELSR1, TRMU, CTSE1, CDPF1, TTC38, PKDREJ, PPARA, WNT7B, ATXN10, FBLN1, RIBC2, SMC1B, FAM118A, UPK3A, NUP50, KIAA0930, PHF21B, ARHGAP8, PRR5-ATHGAP8, ARHGAP8, PRR5, RTL6, PARVG, SHISAL1, PARVB, SAMM50, PNPLA3, PNPLA5, SULT4A1, EFCAB6, MPPED1*.

Tabla S2 Supplemental data. Unsupervised clusters based on deletion size and GFAP.

Variable	*SHANK3* SNVs (Cluster-5)	Cluster-3	Cluster-1	Cluster-2	Cluster-4
Gender	8F/13M	18F/21M	52F/40M	14F/15M	20F/9M
Deletion size (Mb)	-	Mean: 0.126±0.75 (median. 0.086) range 0.34	Mean: 2.73±1.26 (median. 2.83) range 4.35	Mean: 6.11±0.69 (median. 6.4) range 2.12	Mean: 8.28±0.74 (median. 8.07) range 3.05
GFAP	Mean:87.52±50.28 (median. 100)	Mean:78.19±46.65 (median. 67)	Mean:102.64±39.38 (median. 102)	Mean:111.17±37.38 (median. 100)	Mean:125.66±29.33 (median. 130)
Walk before 15 montas	**13/18 (72.2%)**	**21/39 (53.8%)**	18/91 (19.4%)	7/28 (24.1 %)	3/29 (10.3%)
No speech	5/18 (27.7%)	**9/39 (7.7%)**	34/91 (36.6%)	10/28 (34.5%)	17/29 (58.6%)
Some words	8/18 (44.4%)	**7/39 (17.94%)**	39/91 (41.9%)	17/28 (58.6%)	10/29 (34.5%)
Make short sentences	5/18 (27.7%)	**23/39 (58.97%)**	18/91 (19.4%)	1/28 (3.4%)	2/29 (6.9%)
Hypotonía	13/21 (62%)	**21/39 (51.2%)**	69/91 (74.2%)	26/28 (89.7%)	26 /29 (89.7%)
Behavioral problems	20/21 (94.1 %)	32/39 (82.05%)	71/91 (76.3%)	**18/28 (62.1%)**	27/29 (93.1%)
Regression	9/19 (47.4%)	**15/39 (38.46%)**	48/91 (51.6%)	14/29 (48.3%)	13/29 (44.8%)
Seizures	**3/19 (15.8%)**	**8/39 (20.5%)**	27/91 (29%)	10/28 (34.5%)	13/29 (44.8%)
High pain tolerance	15/19 (79%)	23/39 (59%)	62/91 (66.7%)	**17/28 (58.6%)**	23/29 (79.3%)
Reduced transpiration	**5/16 (31.2%)**	25/39 (64.1%)	**44/91 (47.3%)**	17/28 (58.6%)	14/29 (48.3%)
Increased transpiration	**0/16 (0%)**	**1/39 (2.6%)**	6/91 (6.5%)	3/28 (10.3%)	1/29 (3.4%)
Microcephaly	2/18 (11.1%)	11/39 (28.2%)	23/91 (24.7%)	**0/29 (0%)**	3/29 (10.3%)
Macrocephaly	4/18 (22.2%)	**6/39 (15.4%)**	18/91 (19.4%)	12/29 (41.4%)	13/29 (44.8%)
Dolichocephaly	**1/17 (5.9%)**	**4/39 (10.3%)**	18/91 (19.4%)	8/29 (27.6%)	8/29 (27.6%)
Flat face	2/18 (11.1%)	5/39 (12.8%)	11/91 (11.8%)	**2/28 (6.9%)**	8/29 (27.6%)
Epicanthus	**2/18 (11.1%)**	12/39 (30.8%)	**24/91 (25.8%)**	10/29 (34.5%)	8/29 (27.6%)

Strabismus	2/18 (11.1%)	**4/39 (10.3%)**	24/91 (25.8%)	9/28 (31%)	12/29 (41.4%)
Ptosis	**2/17 (11.8%)**	**6/39 (15.4%)**	17/91 (18.3%)	3/28 (10.3%)	8/29 (27.6%)
Deep set eyes	**1/18 (5.5%)**	**7/39 (17.9%)**	17/91 (18.3%)	11/28 (37.9%)	9/29 (31%)
Long eyelids	10/18 (55.6%)	**19/39 (48.7%)**	50/91 (53.8%)	17/28 (58.6%)	21/29 (72.4%)
Full brow	**1/17 (5.9%)**	**10/39 (25.6%)**	39/91 (41.9%)	11/28 (37.9%)	14/29 (48.3%)
Fully/puffy eye	**1/18 (5.5%)**	2/39 (51.1%)	**25/91 (26.9%)**	8/28 (27.6%)	8/29 (27.6%)
Broad nasal bridge	**8/20 (40%)**	29/39 (51.3%)	**47/91 (50.5%)**	16/28 (55.2%)	22/29 (75.9%)
Bulbous nose	**7/18 (38.9%)**	**18/39 (46.2%)**	53/91 (57%)	19/28 (65.5%)	18/29 (62.1%)
Hearing problems	8/19 (42.1%)	**13/39 (33.3%)**	37/91 (39.8%)	16/29 (55.2%)	20/29 (69%)
Fully/puffy cheeks	4/19 (21%)	**3/39 (7.7%)**	24/91 (25.8%)	6/28 (20.7%)	9/29 (31%)
Teeth anomalies	**3/18 (16.7%)**	19/39 (48.7%)	**37/91 (39.8%)**	12/28 (41.4%)	19/29 (65.5%)
Point chin	11/18 (61.1%)	25/39 (64.1%)	**51/91 (54.8%)**	16/28 (55.2%)	17/29 (58.6%)
Syndactyly	**1/18 (5.5%)**	**3/39 (7.7%)**	25/91 (26.9%)	10/28 (34.5%)	13/29 (44.8%)
Dysplastic nails	7/18 (38.9%)	**10/39 (25.6%)**	39/91 (41.9%)	12/28 (41.4%)	15/29 (51.7%)
Large hands	8/17 (47.1%)	**11/39 (28.2%)**	50/91 (53.8%)	20/28 (69%)	20/29 (69%)
Clinodactyly	**1/17 (5.9%)**	**5/39 (12.8%)**	19/91 (20.4%)	4/28 (13.8%)	7/29 (24.1%)
Cardiac anomalies	**1/17 (5.9%)**	6/39 (15.4%)	**14/92 (15.1%)**	5/29 (17.2%)	5/29 (17.2%)
Sleep problems	11/19 (57.9%)	8/39 (20.5%)	24/91 (25.8%)	**3/28 (10.3%)**	10/29 (34.5%)
ophthalmological anomalies	4/17 (23.5%)	**6/39 (15.4%)**	17/91 (18.3%)	9/28 (31%)	9/29 (31%)
Nephro-urological anomalies	**1/17 (5.9%)**	7/39 (17.9%)	**15/91 (16.1%)**	6/28 (20.7%)	14/29 (48.3%)
Anomalías de la línea media	2/18 (11.1%)	3/39 (7.7%)	**6/91 (6.5%)**	2/29 (6.9%)	6/29 (20.7%)
Dermatological problems	4/17 (23.5%)	**6/39 (15.4%)**	18/91 (19.4%)	8/29 (27.6%)	10/29 (24.5%)
Recurrent infection	5/18 (27.8%)	**5/39 (13.6%)**	16/91 (17.2%)	6/28 (20.7%)	3/29 (10.3%)
Hernia	**0/17 (0%)**	**1/39 (2.6%)**	4/91 (4.3%)	4/28 (13.8%)	3/29 (10.3%)
Obesity	**0/17 (0%)**	**0/39 (0%)**	1/91 (1.1%)	1/29 (3.4%)	2/29 (6.9%)
Lymphedema	**0/17 (0%)**	**1/39 (2.6%)**	4/91 (4.3%)	3/28 (10.3%)	10/29 (34.5%)

Gastrointestinal problems	5/18 (27.8%)	6/39 (15.4%)	19/91 (20.4%)	**1/28 (3.4%)**	8/29 (27.6%)
Poor visual contact	9/18 (50%)	25/39 (64.1%)	**46/91 (49.5%)**	15/28 (51.7%)	20/29 (69%)
bit	7/18 (38.9%)	12/39 (30.8%)	36/90 (40%)	**8/28 (27.6%)**	13/29 (44.8%)
High sensitive	12/18 (66.7%)	**11/39 (28.2%)**	30/91 (32.3%)	9/28 (31%)	11/29 (37.9%)
Uncontrolled laughs	7/18 (38.9%)	**11/39 (28.2%)**	32/91 (34.4 %)	10/28 (34.5%)	16/29 (55.2%)
Impulsivity	**9/19 (47.4%)**	21/39 (53.8%)	48/91 (51.6%)	14/28 (48.3%)	**14/29 (48.3%)**
Excessive cries	5/18 (27.8%)	14/38 (35.9 %)	31/91 (33.3%)	**8/28 (27.6%)**	16/29 (55.2%)
Pulling hair	4/17 (23.5%)	**4/39 (10.3%)**	22/91 (23.7 %)	7/28 (24.1%)	9/29 (31%)
pinching	4/17 (23.5%)	**7/39 (17.9%)**	24/91 (25.8%)	6/28 (20.7%)	6/29 (20.7%)
Do not stop crying	4/17 (23.5%)	4/39 (10.3%)	15/91 (16.1%)	**1/29 (3.4%)**	7/29 (24.1%)
Aggressively	2/19 (10.5%)	8/39 (20.5%)	19/91 (20.4%)	6/28 (20.7%)	**3/29 (10.3%)**
Play with tongue	6/18 (33.3%)	16/39 (41%)	**25/91 (26.9%)**	10/28 (34.5%)	11/29 (37.9%)
Abnormal emotional responses	14/18 (77.8%)	24/39 (61.5%)	50/91 (53.8%)	**11/28 (37.9%)**	13/29 (44.8%)
ASD	7/21 (33.2%)	4/39 (10.3%)	11/91 (11.8%)	**2/28 (6.9%)**	5/29 (17.2%)
Slow growth	**0/18 (0%)**	**1/39 (2.56%)**	14/91 (15.1%)	3/29 (10.3%)	4/29 (13.8%)
Accelerated growth	**2/18 (11.1%)**	**8/39 (20.51%)**	28/91 (30.1%)	9/29 (31%)	16/29 (55.2%)

Table S3 Supplemental data. Relationship of categorical variables that show statistical significance between the clusters (CL1-CL4, and *SHANK3* variants; CL-5), and their interrelation between them, using the Chi-square test. a) CL4 *vs.* CL3; b) CL2 *vs.* CL1; c) CL2 *vs.* CL4; d) CL3 *vs.* CL5; e) CL2 *vs.* CL3 *v: value; gl: degrees of freedom; p: p value

**a) Chi-square test**	**Value**	**df**	**Significance (p)(bilateral)**	**Interpretation**
**Ability to make Sentences**	16.59	1	**<.0001 ****	**CL4 has a significantly lower chance of having the ability to make sentences compared to CL3**
**Walk independent before 15 months**	8.466	1	**.004 FeT ***	**CL3 have a much higher chance of walking before 15 months compared to CL4**
**Hypotonia**	10.806	1	**<.0001 FeT ****	**CL3 has a significantly lower chance of having hypotonia compared to CL4**

**Sphincter control**	6.198	**1**	**.015 FeT ***	**CL3 has a significantly higher chance of having sphincter control compared to CL4**
**Toe syndactily**	15.043	1	**<.0001 ****	**CL3 has a significantly lower chance of having toe syndactyly compared to CL4**
**Large and Fleshly hands**	6.482	1	**.011 ***	**CL4 has a significantly higher chance of having large/fleshly hands compared to CL3**
Growth anomalies	8.466	1	**.004 ***	**CL4 has a significantly higher chance of having growth anomalies compared to CL3**
Wide nasal bridge	3.888	1	**.049 ***	**CL4 has a significantly higher chance of having wide nasal bridge compared to CL3**
Prominent/dysplastic ears	5.672		**.049 ***	**CL4 has a significantly higher chance of having prominent/dysplastic compared to CL3**
Nephrourological anomalies	6.482	1	**.011 ***	**CL4 has a significantly higher chance of having nephro-urological anomalies compared to CL3**
Lymphedema	7.657	1	**.010 FeT ***	**CL4 has a significantly higher chance of having Lymphedema compared to CL3**
Macrocephaly	3.890	1	**.054**	**CL3 has a trending lower chance of macrocephaly compared to CL4**
**b) Chi-square test**	**Value**	**df**	**Significance (p)(bilateral)**	**Interpretation**
**Dep set eyes**	4.538	1	**.033 ***	**CL2 have a much higher chance of having deep set eyes compared to CL1**
**Opthalmological anomalies (OA)**	3.938	1	**.047 ***	**CL2 have a much higher chance of having OA compared to CL1**
**Sphincter control**	4.587	**1**	**<.0001 FeT ****	**CL2 has no chance of having sphincter control compared to CL1**
**Obesity**	3.976	1	**.05**	**CL1 has a significantly lower chance of having obesity compared to CL1**
**Lymphedema**	8.596	1	**.008 ****	**CL2 has a significantly higher chance of having lymphedema compared to CL1**
**c) Chi-square test**	**Value**	**df**	**Significance (p)(bilateral)**	**Interpretation**
**Behavior anomalies**	4.035		**.05 FeT ***	**CL4 has a significantly higher chance of having lymphedema compared to CL2**
Nephrourological anomalies	4.047	1	**.044 ***	**CL4 has a significantly higher chance of having nephro-urological anomalies compared to CL2**
d) Chi-square test	Value	df	**Significance (p)(bilateral)**	**Interpretation**

Walk independent before 15 months	4.46		**.049 FeT ***	**CL5 has a significantly higher chance of being able to walk before 15 months compared to CL3 Logistic regression established among 3.36 times**
Ability to make Sentences	3.898	1	**.058**	**CL5 has a trending lower chance of having the ability to make sentences compared to CL3**
Dep set eyes	3.98	1	**.056**	**CL5 has a trending lower chance (no chance) of having deep set eyes compared to CL3**
Full brow	4.538	1	**.054**	**CL5 has a trending lower chance of having full brow compared to CL3**
Teeth anomalies	7.392	1	**.009 FeT ***	**CL3 has a significantly higher chance of having teeth anomalies compared to CL5**
High sensitive	10.046	1	**.02 ***	**CL5 has a significantly higher chance of being high sensitive compared to CL3**
d) Chi-square test	Value	df	**Significance (p)(bilateral)**	
Growth anomalies	6.856	1	**.013 FeT ***	**CL1 has a significantly higher chance of having growth anomalies compared to CL5**
Walk independent before 15 months	22.375	1	**<.0001 FeT ****	**CL5 have a much higher chance of walking before 15 months compared to CL1**
Ability to make Sentences	3.898	1	**.058**	**CL1 has a trending lower chance of having the ability to make sentences compared to CL5**
Full brow	8.338	1	**.004 FeT ***	**CL5 has a trending lower chance of having full brow compared to CL1**
Full/puffy eyelids	6.509	1	**.010 FeT ***	**CL5 has a significantly lower chance (no chance) of having full/puffy eyelids compared to CL1**
Teeth anomalies	5.02	1	**.027 FeT ***	**CL1 has a significantly higher chance of having teeth anomalies compared to CL5**
Toe syndactily	6.509	1	**.010 FeT ***	**CL5 has a significantly lower chance (no chance) of having toe syndactyly compared to CL1**
Sphincter control	14.300	1	**<.0001 FeT ****	**CL5 has a significantly higher chance of having sphincter control compared to CL1**
Sleeping anomalies	6.294	1	**.012 ***	**CL5 has a significantly higher chance of having sleeping anomalies compared to CL1**
Poor visual contact	4.904	1	**.032 FeT ***	**CL5 has a significantly higher chance of having poor visual contact compared to CL1**
High sensitive	7.689	1	**.006 ***	**CL5 has a significantly higher chance of being high sensitive compared to CL1**
e) Chi-square test	Value	df	**Significance (p)(bilateral)**	**Interpretation**

Macrocephaly	3.987	1	**.051**	**CL3 has a trending lower chance of macrocephaly compared to CL2**

FeT, Fisher´s exact Test.*p-value less than 0.05 indicates a significant difference.

Table S4 Supplemental data. Relationship of categorical variables that show statistical significance between the deletion groups, and *SHANK3* variants, and their interrelation between them, using the Chi-square test. a) Group4 *vs.* Group3; b) Group5 *vs.* Group3; c) Group4 *vs.* Group5; a) Group1+2 *vs.* Group3*v: value; gl: degrees of freedom; p: p value.

**Logistic regression or Chi square tests**				
**a)**	**Coefficient**	**p-value**	**OR (CI)**	**Interpretation**
**Alteration of Growth PC>95% or PC<3%**	-1.764	**.022 ***	0.171[0.038, 0.777]	**Individuals with growth anomalies are less likely to belong to the category of group 3**
**Walk independent before 15 months**	2.12	.00041 *	8.37[2.58, 27.20]	**Individuals with the ability to walk independent before 15 months are approximately 8.37 times more likely to fall into group 3 compared to group 4**
**Epicanthal folds**	4.211	**.044 FeT *** (1, df)		**Group 3 has a significantly lower chance of having epicanthus compared to Group 4**
**Deep set eyes**	4.571	**.045 FeT *** (1, df)		**Group 3 has no chance of having deep set eyes compared to Group 4**
**Full brow**	-2.33	.026 * (1, df)	0.97[0.013, 0.753]	**The results show that having full eyebrows is significantly associated with a lower probability of belonging to the group3.**
**Full/puffy eyelids**	5.133	**.024 FeT *** (1, df)		**Group 3 has no chance of having full/puffy eyelids compared to Group 4**
**Teeth anomalies**	-1.83	.017 *	0.16[0.035, 0.725]	**The presence of spaced teeth/malocclusion is significantly associated with a lower probability of belonging to the group 3**
**Ability to make Sentences**	4.102	**.038 *** (1, df)		**Group 3 has a significantly lower chance of having the ability to make sentences compared to Group 4**
**Toe syndactily**	6.991	**.007 FeT **** (1, df)		**Group 3 has no chance of having toe syndactyly compared to Group 4**
**Sphincter control**	1.72	.0013 *	5.58[1.95, 9.31]	**Sphincter control are significantly associated with a higher probability of belonging to the group 3**
**Sleeping problems**	1.21	0.021 *	3.34[1.20, 15.92]	**Group 3 has a significantly higher chance of having sleeping anomalies compared to Group 4**
**High sensitive (touch, caress, skim)**	1.50	0.0076 *	4.46[1.20, 13.39]	**High sensitivity is significantly associated with a higher probability of belonging to the group 3**

**Logistic regression or Chi square tests**				
**b)**	**Coefficient**	**p-value**	**OR (CI)**	**Interpretation**
**Alteration of Growth PC>95% or PC<3%**	**-1.92**	**.018 ***	**0.146[0.03, 0.716]**	**Individuals with growth anomalies are less likely to belong to the category of group 3**
**Walk independent before 15 months**	**2.54**	**0.0002 ***	**12.64[3.31, 48.21]**	**Individuals with the ability to walk independent before 15 months are approximately 112.64 times more likely to fal into group 3 compared to group 5**
**Seizures**	**-1.65**	**.043 ***	**0.193[0.039, 0.947]**	**The presence of seizures is significantly associated with a lower probability of belonging to the group 3**
**Deep set eyes**	**6.383**	**.012 FeT * (1, df)**		**Group 3 has no chance of having deep set eye compared to Group 5**
**Full brow**	**-2.33**	**0.025 ***	**0.09[0.011, 0.743]**	**The results show that having full eyebrows is gnificantly associated with a lower probabilit of belonging to the group 3.**
**Full/puffy eyelids**	**4.079**	**.05 FeT * (1, df)**		**Group 3 has no chance of having full/puffy eyelids compared to Group 5**

**Logistic regression or Chi square tests**				
**c)**	**Coefficient**	**p-value**	**OR (CI)**	**Interpretation**
**Large/fleshly hands**	3.976	.046 * (1,.df)		**Group 4 has a significantly higher chance of having individuals with large/fleshly hands compared to Group 5**
**Sphincter control**	4.210	0.040 * (1.df)		**Group 4 has a significantly higher chance of having individuals with sphincter control compared to Group 5**

**Chi-square test**				
**d)**	**Value**	**df**	**Significance (p)(bilateral)**	**Interpretation**
**Walk independent before 15 months**	16.261	1	**.0001 ***	**SNV Group has a much higher chance of walking before 15 months compared to deletions**
**Epicanthal folds**	5.14	1	**.046 FeT ***	**deletions has a significantly higher chance of having epicanthus compared to SNVs group**
**Full brow**	7.61	1	**.007 FeT ***	**SNV Group has a much lower chance of having full brown compared to deletions**
**Full/puffy eyelids**	7.82	1	**.026 FeT ***	**SNV Group has no chance of having full/puffy eyelids compared to deletions**
**Teeth anomalies**	7.87	1	**.005 FeT ***	**SNV Group has a much lower chance of having teeth anomalies compared to deletions**

**Chi-square test**				
**Toe syndactily**	6.18	1	**.008 FeT ***	**SNV Group has no chance of having toe syndactyly compared to deletions**
**Sphincter control**	4.71	**1**	**.0001 ***	**SNV Group has a significantly higher chance of having a sphincter control compared to deletions**
**Sleeping problems**	6.67	1	**.016 ***	**SNV Group has a significantly higher chance of having a sleeping problems contact compared to deletions**
**Poor visual contact**	4.24	1	**.043 FeT ***	**SNV Group has a significantly higher chance of having a poor visual contact compared to deletions**
**High sensitive (touch, caress, skim)**	9.77	1	**.002***	**SNV Group has a significantly higher chance of being high sensitive compared to deletions**
**Be emotional**	3.67	1	**.047 FeT***	**SNV Group has a significantly higher chance of being so emotional compared to deletions**

FeT, Fisher´s exact Test.*p-value less than 0.05 indicates a significant difference.

Tabla S5 Supplemental data. Gene clustering with GFAP: a) score 10-20. b) Gene clustering with GFAP score < 10. Genomic coordinates in GRCh38 (hg38).

a)

**GEN**	**COORDINATES**	**SCORE (AU)**
BIK	chr22: 43,110,750-43,129,712	12
SAMM50	chr22: 43,955,442-44,010,531	12
PARVB	chr22: 43,999,211-44,172,939	12
KIAA0903	chr22: 45,192,244-45,240,894	11
FAM118A	chr22: 45,308,968-45,341,955	16
SMC1B	chr22: 45,344,063-45,413,619	19

ATXN10	chr22: 45,671,798-45,845,307	12
TTC38	chr22: 46,267,961-46,294-008	12
CERK	chr22: 46,684,410-46,738,252	12
CRELD2	chr22: 49,918,167-49,927,540	12
IL17REL	chr22: 49,991,811-50,012,765	12
TTLL8	chr22: 50,018,575-50,058,298	11
MLC1	chr222: 50,059,391-50,085,426	12
MOV10L1	chr22: 50,090,006-50,161,690	12
PANX2	chr22: 50,170,731-50,180,295	11
TRABD	chr22: 50,185,913-50,199,598	12
TUBGCP6	chr22: 50,217,689-50,245,023	12
HDAC10	chr22: 50,245,183-50,251,405	12
MAPK12	chr22: 50,245,450-50,261,716	12
MAPK11	chr22: 50,263,713-50,270,767	12
DENND6B	chr22: 50,309,030-50,327,012	11
PPP6R2	chr22: 50,343,304-50,445,090	17
ADM2	chr22: 50,481,543-50,486,440	11
MIOX	chr22: 50,486,784-50,490,648	12
LMF2	chr22: 50,502,949-50,507,702	11
NCAPH2	chr22: 50,508,224-50,524,780	17
SCO2	chr22: 50,523,568-50,526,461	12
TYMP	chr22: 50,525,752-50,530,032	17
KLHDC7B	chr22: 50,545,899-50,551,023	12
SYCE3	chr22: 50,551,112-50,562,919	18
CPT1B	chr22: 50,568,861-50,578,465	17
CHKB-CPT1B	chr22: 50,568,869-50,582,965	10
ARSA	chr22: 50,622,754-50,628,173	11
ACR	chr22: 50,738,196-50,745,339	12

		Average: 12.823

b)

GEN	COORDINATES	SCORE (AU)
MCAT	chr22: 43,132,209-43,143,398	2
TSPO	chr22: 43,166,622-43,187,134	1
EFCAB6	chr22: 43,824,509-43,812,337	2
PNPLA5	chr22: 43,879,678-43,892,013	2
PNPLA3	chr22: 43,923,792-43,964,488	2
PARVG	chr22: 44,172,956-44,219,533	2
SHISAL1	chr22: 44,243,665-44,312,951	1
RTL6	chr22: 44,492,583-44,498,233	1
PRR5-ARHGAP8	chr22: 44,702,233-44,862,706	1
ARHGAP8	chr22: 44,752,559-44,862,788	1
UPK3A	chr22: 45,284,949-45,295,874	1
RIBC2	chr22: 45,413,693-45,432,509	1
CDPF1	chr22: 46,244,011-46,250,311	2
PKDREJ	chr22: 46,255,663-46,26,343	2
GTSE1	chr22: 46,296,870-46,330,810	2
TRMU	chr22: 46,330,875-46,357,340	2
TAFA5	chr22: 48,489,553-48,850,912	3
SELENOO	chr22: 50,201,011-50,217,616	1
CIAMP1B	chr22: 50,529,710-50,532,506	1
CHKB	chr22: 50,578,959-50,601,455	2
		Average: 1.6
